# Transcriptome Profiling of Spinal Muscular Atrophy Motor Neurons Derived from Mouse Embryonic Stem Cells

**DOI:** 10.1371/journal.pone.0106818

**Published:** 2014-09-05

**Authors:** Miho Maeda, Ashlee W. Harris, Brewster F. Kingham, Casey J. Lumpkin, Lynn M. Opdenaker, Suzanne M. McCahan, Wenlan Wang, Matthew E. R. Butchbach

**Affiliations:** 1 Center for Applied Clinical Genomics, Nemours Biomedical Research, Nemours Alfred I. duPont Hospital for Children, Wilmington, Delaware, United States of America; 2 Center for Pediatric Research, Nemours Biomedical Research, Nemours Alfred I. duPont Hospital for Children, Wilmington, Delaware, United States of America; 3 Bioinformatics Core Facility, Nemours Biomedical Research, Nemours Alfred I. duPont Hospital for Children, Wilmington, Delaware, United States of America; 4 Department of Biological Sciences, University of Delaware, Newark, Delaware, United States of America; 5 Sequencing and Genotyping Center, University of Delaware, Newark, Delaware, United States of America; 6 Center for Translational Cancer Research, University of Delaware, Newark, Delaware, United States of America; 7 Department of Pediatrics, Thomas Jefferson University, Philadelphia, Pennsylvania, United States of America; National University of Singapore, Singapore

## Abstract

Proximal spinal muscular atrophy (SMA) is an early onset, autosomal recessive motor neuron disease caused by loss of or mutation in *SMN1* (*survival motor neuron 1*). Despite understanding the genetic basis underlying this disease, it is still not known why motor neurons (MNs) are selectively affected by the loss of the ubiquitously expressed SMN protein. Using a mouse embryonic stem cell (mESC) model for severe SMA, the RNA transcript profiles (transcriptomes) between control and severe SMA (*SMN2^+/+^;mSmn^−/−^*) mESC-derived MNs were compared in this study using massively parallel RNA sequencing (RNA-Seq). The MN differentiation efficiencies between control and severe SMA mESCs were similar. RNA-Seq analysis identified 3,094 upregulated and 6,964 downregulated transcripts in SMA mESC-derived MNs when compared against control cells. Pathway and network analysis of the differentially expressed RNA transcripts showed that pluripotency and cell proliferation transcripts were significantly increased in SMA MNs while transcripts related to neuronal development and activity were reduced. The differential expression of selected transcripts such as *Crabp1*, *Crabp2* and *Nkx2.2* was validated in a second mESC model for SMA as well as in the spinal cords of low copy *SMN2* severe SMA mice. Furthermore, the levels of these selected transcripts were restored in high copy *SMN2* rescue mouse spinal cords when compared against low copy *SMN2* severe SMA mice. These findings suggest that SMN deficiency affects processes critical for normal development and maintenance of MNs.

## Introduction

Spinal muscular atrophy (SMA) is an autosomal recessive, early-onset neurodegenerative disorder characterized by the degeneration of α-motor neurons (MNs) in the anterior horn of the spinal cord which leads to progressive muscle weakness and atrophy [1]. SMA is a leading genetic cause of infant death worldwide with 1 in 5000–10,000 children born with the disease [2], [3] and a carrier frequency of 1∶25–50 [4]–[7]. SMA results from the loss or mutation of the *SMN1* (*survival motor neuron 1*) gene on chromosome 5q13 [8]. There is an inverted duplication of *SMN1* in humans called *SMN2* [9], [10]. The duplication of *SMN1* only occurs in humans. Within *SMN2*, there is a C-to-T transition in an exonic splice enhancer of exon 7 that results in the vast majority (about 80–90%) of *SMN2* mRNAs to lack exon 7 (SMNΔ7). SMNΔ7 is not fully functional and prone to degradation [11], [12]. *SMN2* can, however, provide some fully functional SMN protein. The copy number of *SMN2* modifies the severity of SMA phenotype in humans [13]–[20]. Although the genetics underlying SMA are known, the mechanisms leading to the disease are poorly understood.

SMN is a ubiquitously expressed protein that facilitates the assembly of ribonucleoproteins (RNPs) [21]. The assembly of U-type small nuclear RNPs (snRNPs) is developmentally regulated in many cell types [22]. Knockdown of snRNP assembly in zebrafish results in degeneration of motor neuron axons [23]. snRNP assembly is defective in SMN-deficient SMA cells [24]. However, this function of SMN in U-type snRNP assembly is required by all cells. So why are MNs primarily affected in SMA given this ubiquitous function? Gabanella et al. show that snRNP assembly is defective in tissues from mouse models for SMA and that the extent of reduced snRNP assembly correlates with phenotypic severity of these SMA mice [22]. Furthermore, snRNP assembly is more markedly affected by Smn deficiency in SMA mouse neural tissues than in other tissues like the kidney. This enhanced sensitivity of neurons to deficits in snRNP assembly may explain why MNs are primarily affected in SMA.

In addition to its role in snRNP biogenesis, SMN may also have functions which are unique to MNs. SMN associates with cytoskeletal elements in MN dendrites and axons within the rat spinal cord [25]. Knockdown of *Smn* expression in zebrafish embryos using morpholino antisense oligonucleotides leads to reduced axonal outgrowth and axonal pathfinding defects [26]. Ectopic overexpression of wild-type SMN in these *Smn*-knocked down zebrafish embryos restores normal axonal growth and pathfinding. Moreover, axonal defects in *Smn*-knocked down zebrafish embryos are corrected by overexpression of mutant SMNs which are incapable of snRNP assembly [27]. The core protein components of snRNPs, the Sm proteins, do not colocalize with SMN in neuronal processes [28]. Taken together, these observations suggest that SMN may have a unique function in neurons that is independent of snRNP biogenesis. Several studies have suggested that SMN may play a role in axonal trafficking. SMN has been shown to interact with the heterogeneous nuclear ribonucleoprotein-R (hnRNP-R) [29]. hnRNP-R binds to the 3′-untranslated region (3′-UTR) of β-actin mRNA [30]. Transport of β-actin mRNA and protein into growth cones has been shown to be essential in developing neurites [31]. Reduced expression of *Smn* in mouse MNs results in reduced axonal outgrowth and trafficking of β-actin mRNA along axons [30]. Conditional knockout of β-actin in mouse MNs, however, does not affect the morphologies of MN neurites suggesting that β-actin mRNA transport along axons may not be essential for MN axon development [32]. Although neuron-specific roles of SMN have been proposed, further studies are needed to determine how these neuron-specific roles are involved in the pathogenesis of SMA.

Mouse models have been instrumental in studying the function of SMN and how this function is disrupted in SMA. Mice, like all animals except humans, have only one SMN gene (*mSmn*) that is equivalent to *SMN1*. Homozygous disruption of *mSmn* in mice is embryonically lethal [33]. Transgenic overexpression of *SMN2* rescues the embryonic lethality of *mSmn* deficiency [34], [35]. *mSmn* nullizygous mice that also harbor few copies of the *SMN2* transgene (i.e. 1 or 2) develop a severe motor phenotype resembling SMA and die within 7 days after birth [34], [35]. Increasing the *SMN2* copy number in these *mSmn* nullizygous mice improves the survival and phenotype of these SMA mice; in fact, expression of 8–16 copies of *SMN2* fully rescues the SMA phenotype in these mice [34], [36]. Patients who have been identified genetically as SMA—i.e. loss of *SMN1*—are phenotypically normal when they carry at least 5 copies of *SMN2* [16]. Thus, *SMN2* expression modifies the phenotypic severity of SMA in mice as well as in man and makes *SMN2* a target for the development of SMA therapeutics.

The low copy *SMN2* SMA mouse phenotypically resembles type I SMA in humans [34]. The short lifespan as well as the low frequency of pups that survive past birth limit their use for mechanistic studies; therefore, an *in vitro* model would be useful for such studies. Murine embryonic stem cells (mESCs) are able to differentiate into spinal neural progenitor cells and then into MNs through exposure to the morphogens retinoic acid (RA) and Sonic hedgehog (Shh) [37]. Motor neurons differentiated from mESCs were found to generate action potentials and developed axons and synapses when co-cultured with muscle cells [38]. mESC lines have been established for low copy *SMN2* severe SMA mice also harboring a MN-specific reporter construct (HB9:eGFP) [39]. When these SMA mESCs are directed to differentiate into MNs, they start dying after the differentiation process [39]. MNs derived from SMA mESCs can, therefore, potentially provide important insights into the pathogenesis of SMA.

In this study, we will use cultured MNs derived from SMA mESCs to determine how reduced levels of the ubiquitously expressed protein SMN result in selective MN death in SMA. Previous studies have used cDNA microarrays to identify differentially expressed mRNAs in SMA mouse whole spinal cords and in primary MN cultures [40]–[42]. Microarrays can only identify known RNA transcripts which limits their utility for comprehensively characterizing transcriptomes. Massively parallel RNA sequencing, commonly known as RNA-Seq [43], is a recently developed deep-sequencing technology used for transcriptome profiling [44]. RNA-Seq directly reads the sequences of the cDNA pool which results in a very low background signal as compared to the indirect method of measuring hybridization intensity used in microarray analysis. Since RNA-Seq directly reads cDNA sequences, novel transcripts and isoforms can be identified. In this study, we use RNA-Seq to annotate and compare the transcriptome profile of MNs derived from severe SMA mESCs with those derived from normal mESCs. Analysis of over-represented biological pathways and networks revealed that SMA mESC-derived MNs have increased expression of RNA transcripts related to pluripotency and reduced expression of neuronal development and function RNA transcripts. This study provides new insights into the molecular consequences of SMN deficiency in MNs and identifies novel targets for the development of neuroprotective therapeutics.

## Materials and Methods

### Ethics Statement

All animal experiments were conducted in accordance with the protocols described in the National Institutes of Health *Guide for the Care and Use of Animals* and were approved by the Nemours Biomedical Research Institutional Animal Care and Use Committee.

### Embryonic Stem Cell Culture

Two different types of mESC lines were used for these experiments. The first set of mESC lines—Hb9 and A2—were provided by Dr. Lee L. Rubin and were derived from either wild-type (*SMN2^+/+^;mSmn^+/+^*) or SMA (*SMN2^+/+^;mSmn^−/−^*) mice expressing the HB9:eGFP reporter construct [39]. The mice used to establish these mESC lines were generated by interbreeding the low-copy *SMN2* carrier mouse (FVB.Cg-Tg(SMN2)89Ahmb*Smn^tm1Msd^*/J) with HB9:eGFP (*mHB9:Gfp1b*) transgenic mouse [37], [45]. The second set of mESC lines—C4 and E2—were provided by Dr. Douglas Kerr [46]. These lines were derived from wild-type and SMA mice, respectively, and do not harbor a motor neuron-specific marker gene.

mESCs were grown as previously described [46], [47]. Briefly, mESCs were grown on a primary mouse embryonic fibroblast feeder layer (Millipore, Billerica, MA, USA) in 10 cm tissue culture dishes. Cells were cultured with medium containing DMEM supplemented with 15% fetal bovine serum (Stem Cell Technologies, Vancouver, BC, Canada), 1% GlutaMax-I (Life Technologies, Carlsbad, CA, USA), 1% MEM non-essential amino acids (Millipore), 1% nucleosides (Millipore), 0.1 mM β-mercaptoethanol (Millipore), 1% penicillin/streptomycin (Life Technologies) and 10 ng/mL murine leukemia inhibitory factor (LIF; Millipore).

### ES Cell Differentiation into MNs

**Figure 1:**
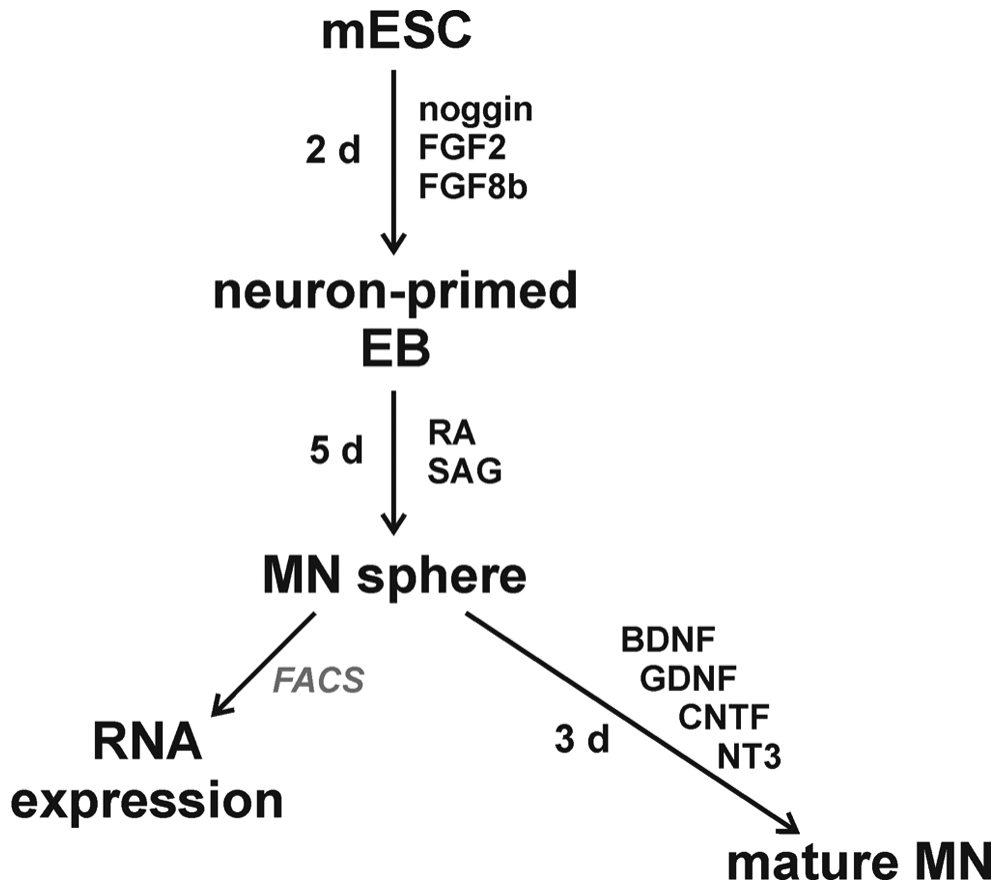
Outline for the differentiation of mouse embryonic stem cells (mESCs) into motor neurons (MNs). mESCs were first converted into neuron-primed embryoid bodies (EBs) by treatment with noggin, fibroblast growth factor 2 (FGF2) and FGF8b for 2 days. The neuron-primed EBs were then directed to differentiate into MN spheres by treatment with retinoic acid (RA) and Smoothened agonist (SAG) for 5 days. The MNs were then dissociated from the spheres and then either isolated using fluorescence-activated cell sorting (FACS) for subsequent RNA isolation or allowed to mature for 3 days with a MN maturation cocktail containing brain-derived neurotrophic factor (BDNF), glial cell-derived neurotrophic factor (GDNF), ciliary neurotrophic factor (CNTF) and neurotrophin-3 (NT3).

mESCs were differentiated into motor neurons (MNs) as outlined in **Figure 1** [46], [47]. Briefly, mESCs were dissociated with 0.25% trypsin/EDTA and placed into a T75 cell culture flask coated with 0.1% gelatin (Stem Cell Technologies). Fibroblasts were allowed to re-attach to the flask. The floating mESCs were collected and plated into a 10 cm petri dish containing 10 mL of Neural Differentiation Medium (DMEM supplemented with 15% FBS, 1% nonessential amino acids, 1% GlutaMax-I, 1% penicillin/streptomycin, 1 mM monothioglycerol (Sigma-Aldrich), 50ng/ml noggin (Life Technologies), 20 ng/mL basic fibroblast growth factor (FGF2; Life Technologies) and 20 ng/mL fibroblast growth factor-8b (FGF-8b; Life Technologies)). Media was replaced daily. After two days, embryoid bodies (EBs) were re-suspended in MN differentiation medium (NITSf; basal medium A (Stem Cell Technologies) supplemented with 10% Knockout SR (serum replacement; Life Technologies), 1% N-2 supplement (Life Technologies), 1% ITS-B supplement (Stem Cell Technologies), 1% ascorbic acid (Stem Cell Technologies), 1% penicillin/streptomycin, 0.1% mM β-mercaptoethanol, 0.5% GlutaMax-I, 30% D-glucose (Sigma-Aldrich), 20 µg/mL heparin (Sigma-Aldrich) and 50 µg/mL fibronectin (Stem Cell Technologies)) in the presence of 1 µM RA (Sigma-Aldrich) and 1 µM Smoothened agonist (SAG; Millipore). Media was replaced daily for 5 days when EBs were collected, washed with phosphate-buffered saline (PBS) and dissociated in Accumax (Millipore). Cell aggregates were filtered through a BD cell strainer and single cells were then suspended in NITSf medium supplemented with 10 ng/mL each of brain-derived neurotrophic factor (BDNF; R&D Systems), glial cell-derived neurotrophic factor (GDNF; R&D Systems), ciliary neurotrophic factor (CNTF; R&D Systems) and neurotrophin-3 (NT-3; R&D Systems). The cells were plated at a concentration of 1×10^5^ cells per well in a 24 well plate that contained coverslips coated with 100 µg/mL poly-DL-ornithine hydrobromide (Sigma-Aldrich), 2 µg/mL laminin (Millipore) and 300 µg/mL Matrigel Matrix Basement Membrane (BD Bioscience, San Jose, CA, USA).

### Animals

Spinal cords were collected from two different mouse models for SMA: the severe low copy *SMN2* SMA (*SMN2(Ahmb89)^+/+^;mSmn^−/−^*) and the high copy *SMN2* rescue (*SMN2(Ahmb(566)^+/+^;mSmn^−/−^*) mice [34]. Both mouse lines are available from Jackson Laboratories (Bar Harbor, ME; stock numbers 005024 and 008026, respectively). To obtain low copy *SMN2* SMA mice, carrier mice ((*SMN2(Ahmb89)^+/+^;mSmn^+/−^*) were interbred to generate SMA, carrier and control (*SMN2(Ahmb89)^+/+^;mSmn^+/+^*) progeny. Since the high copy *SMN2* rescue mice are phenotypically normal [34], the necessary mice were generated from interbreeding rescue mice.

Spinal cords were collected at 2 time points: embryonic day 13.5 (e13.5) and postnatal day 3 (PND03; with the day of birth being defined as PND01). For collecting e13.5 samples, timed-pregnant dams (as assessed by the presence of a vaginal plug) were euthanized at e13±0.5 and the spinal cords were rapidly dissected from the embryos, snap-frozen and stored at −80°C until RNA isolation. Additional tissues were harvested from each embryo for genotyping. For postnatal samples, pups were euthanized and the spinal cords were rapidly dissected from the pups, snap-frozen and stored at −80°C until RNA isolation. Tail biopsies were also taken for genotyping. All embryonic and postnatal samples were genotyped as described previously [34], [48].

### Immunofluorescence

Cells grown on coverslips were washed with PBS. Cells were fixed with 4% paraformaldehyde (Sigma-Aldrich) in PBS for 20 min. Cells were rinsed with PBS+ (0.1% saponin (Sigma-Aldrich) and 0.02% NaN_3_ (Sigma-Aldrich) in PBS, pH 7.4) and blocked with PBS+BSA (20 mg/mL bovine serum albumin (BSA; Sigma-Aldrich) in PBS+) for 30 min. Cells were then incubated overnight at 4°C with mouse anti-Hb9 (1∶10; clone 81.5C10, Developmental Studies Hybridoma Bank (DSHB), Iowa City, IA, USA), mouse anti-Tuj1 (1∶1000; Covance, Princeton, NJ, USA), mouse anti-Islet-1 (1∶50; clone 40.2D6, DSHB), mouse anti-nestin (1∶500, Millipore), or mouse anti-NeuN (1∶200, Millipore) in PBS+BSA supplemented with rabbit anti-GFP (1∶2000, Rockland Immunochemicals, Gilbertsville, PA, USA). After three washes with PBS+, cells were incubated for 1 hr at room temperature with Alexa Fluor 594 goat anti-mouse IgG (Life Technologies) and Alexa Fluor 488 goat anti-rabbit IgG (Life Technologies) in PBS+BSA. Cells were washed three times with PBS+ and incubated with 100 ng/mL Hoechst 33342 (Life Technologies) in ddH_2_O for 10 min and rinsed with ddH_2_O before mounting in Immu-Mount (Shandon-Lipshaw). Images were obtained using a Leica TCS SP5 confocal microscope.

### Immunoblot Analysis

Cells were pelleted and lysed in a lysis buffer containing 20 mM Tris-HCl, pH 7.4, 150 mM sodium chloride (NaCl), 1% Triton X-100, 1 mM ethylenediaminetetraacetic acid (EDTA), 1 mM ethylene glycol-bis(2-aminoethylether)-N,N,N′,N′-tetraacetic acid (EGTA), 1 mM sodium glycerolphosphate, 2.5 mM sodium pyrophosphate, 100 mM sodium fluoride (NaF), 10% glycerol, 1 mM sodium orthovanadate, 1 mM phenylmethylsulfonylfluoride (PMSF), 5 µg/mL aprotinin and 2 µg/mL leupeptin. The lysates were sonicated and centrifuged at 13,200 rpm for 15 min. Protein concentration of the supernatants were analyzed with Pierce BCA Protein Assay Kit (Thermo Scientific, Rockford, IL, USA). 50 µg of protein per lane were electrophoretically separated in a 10% polyacrylamide gel containing 0.1% SDS and transferred onto a Hybond-P PVDF membrane (GE Healthcare, Piscataway, NJ, USA). The blots were blocked in 5% nonfat milk in Tris-buffered saline with 0.1% Tween-20 (TBS-T) for 1 hr. The blots were then incubated overnight at 4°C with mouse anti-SMN antibody (1∶5000, BD Biosciences) diluted in 3% BSA in TBS-T. The blots were then washed three times with TBS-T and then incubated for 1 hr with horseradish peroxidase (HRP)-conjugated sheep anti-mouse IgG (1∶2500, GE Healthcare) diluted in 3% BSA in TBS-T. The protein on the membrane was detected using a chemiluminescence buffer containing 1.25 mM luminol (Sigma-Aldrich), 198 µM p-coumaric acid (Sigma-Aldrich) and 0.00915% H_2_O_2_ (Sigma-Aldrich) in 100 mM Tris-HCl, pH 8.5. The membrane was then stripped with Re-Blot Plus Strong Solution (Millipore) and reprobed using rabbit anti-glyceraldehyde-3-phosphate dehydrogenase (GAPDH; 1∶20,000, Sigma-Aldrich) as primary antibody and HRP-conjugated goat anti-rabbit IgG (1∶2500, Rockland Immunochemicals) as secondary antibody.

### Fluorescence-Activated Cell Sorting

Dissociated ES cells were filtered through a cell strainer, suspended in DMEM with 20 µg/mL of propidium iodide (PI) and sorted using a 488 nm laser attached to either a MoFlo cell sorter (Coulter; Kimmel Cancer Center, Thomas Jefferson University) or the FACSAria II sorter (BD Biosciences; Center for Translational Cancer Research, University of Delaware). Cells were passed through a 100 µm nozzle tip at a speed of approximately 12,500 events per second. Embryonic stem cells from nonGFP-expressing, wild-type mice described previously [46] were used as a negative control to set the cutoff for background fluorescence. Cells that were GFP positive, PI negative were collected in a 5 mL round bottom test tube (BD Biosciences, Franklin Lakes, NJ, USA) with PBS supplemented with 60% FBS. Approximately a million cells were collected per sample. Sorted cells were centrifuged, PBS was removed and pellets were snap frozen for RNA isolation.

### RNA Isolation

Total RNA was isolated from cells using the RNeasy mini kit (QIAGEN, Germantown, MD, USA) with an additional DNase I (QIAGEN) digestion step to remove any genomic DNA contamination. The concentration of the purified RNA was determined by a ND-1000 NanoDrop the spectrophotometer (NanoDrop Technologies, Wilmington, DE). RNA integrity was assessed by the Agilent Technologies 2100 Bioanalyzer.

### RNA-Seq

1 µg of RNA from each sample (n = 3/genotype) was collected and sent to the Sequencing and Genotyping Center at the Delaware Biotechnology Institute in the University of Delaware for RNA-Seq library preparation and sequencing. The cDNA library was prepared using the TruSeq RNA Sample Prep Kit (Illumina, San Diego, CA) according to the manufacturer's directions. The samples were then clustered and sequenced on an Illumina HiSeq 2500. Deep sequencing was performed on triplicates for each cell line (total six samples) for a 50 cycle single end run. The raw sequence data have been deposited in the NCBI Gene Expression Omnibus (GEO) [49] under accession number GSE56284.

### RNA-Seq Data Analysis

RNA-Seq reads were assessed for quality control with FastQC (version 0.10.1; Babraham Bioinformatics, Cambridge, UK). Adapter sequences and poly-A tails were removed from sequencing reads with CutAdapt (MIT, Cambridge, MA) [50] and were confirmed by FastQC. Reads were mapped to a reference mouse transcriptome and genome (NCBI m37) using TopHat (version 2.04; [51]). The reference genome and annotation was obtained from Ensembl (http://www.ensembl.org). Transcripts were assembled and transcript abundances were measured as fragments per kilobase of exon per million fragments mapped (FPKM) by Cufflinks [52]. Cuffdiff [53] was then used to determine differential expression. Differential expressed genes were plotted using the R package CummeRbund [53].

### Gene Pathways and Networks Analysis

Identification of biological pathways and networks affected by Smn deficiency in mESC-derived MNs was completed using Ingenuity Pathway Analysis (IPA build version 261899; Ingenuity Systems, Inc., Redwood City, CA). The list of differentially expressed transcripts generated from Cuffdiff was divided into upregulated and downregulated lists of transcripts and were imported into IPA. Biological function and canonical pathways were determined to be over-represented using the Fisher exact test with a false discovery rate (FDR) correction (p≤0.05). A set of interacting biological networks was generated in IPA from the upregulated and downregulated transcript lists. A network score was computed based on the likelihood that the focus transcripts specifically belong to this network as opposed to being in the network by chance. Based on other published work [54], only those networks with scores greater than or equal to 15 were considered relevant for further analysis.

The Upstream Regulator Analysis (URA) algorithm of IPA allows for the identification of upstream regulators—transcription factors, signaling molecules and drugs—which can affect gene expression [55]. URA was completed on upregulated and downregulated transcripts and list of putative upstream regulators was provided. Each upstream regulator was assigned an activation z-score; upstream regulators were considered as being activated or inhibited if their activation z-scores were either greater than or equal to 2.0 for activated regulators or less than or equal to −2.0 for inhibited regulators.

### cDNA Synthesis and Quantitative Reverse Transcriptase-Polymerase Chain Reaction (qRT-PCR)

cDNA was prepared from 0.5–1 µg total RNA using the iScript cDNA Synthesis Kit (Bio-Rad, Hercules, CA, USA) as per manufacturer's instructions. The cDNA—diluted 200–400 fold—was amplified via RT-PCR using the QuantiTect SYBR Green PCR kit (QIAGEN). The target primers listed in **Table 1** were synthesized by Integrated DNA Technologies (Coralville, IA) and primers for the reference transcripts *β-glucuronidase* (*Gusb*), *phosphoglycerate kinase 1* (*Pgk1*) and *ribosomal protein L13a* (*Rpl13a*) were purchased from Real Time Primers LLC (Elkins Park, PA). Quantitative PCR was performed in a 384 well plate on a 7900 HT Fast Real-Time PCR system (Applied Biosystems, Foster City, CA). Each sample was assayed in triplicate. Relative transcript levels were calculated using the comparative C_t_ (2^−ΔΔCt^) method [56] where ΔC_t_ is defined as the difference between the C_t_ for the target transcript and the C_t_ for the geometric mean of *Gusb*, *Pgk1* and *Rpl13a* [57] and ΔΔC_t_ is defined as the difference between the ΔC_t_ for the SMA sample and the ΔC_t_ for the control sample.

**Table 1:** Sequences of primers used for qRT-PCR.

Gene Name	Primers (5′->3′)	Ampicon size (bp)
*Crabp1*	(F) CTTCAAGGTCGGAGAGG (R) GGAACAAGCTGGCCACC	238
*Crabp2*	(F) GGAGATTAACTTCAAGATCGGGGA (R) GCTAGTTTGTAAGATGGACGTGGG	321
*Isl1*	(F) CGGAGAGACATGATGGTGGTT (R) GGCTGATCTATGTCGCTTTGC	109
*Nkx2.2*	(F) CCTCCCCGAGTGGCAGAT (R) GAGTTCTATCCTCTCCAAAAGTTCAAA	74
*Pla2g1b*	(F) CAGGCGCTGCTGCACACAG (R) GTCTAAGTCGTCCACTGGGGTGC	158
*Smn1*	(F) TGCTCCGTGGACCTCATTTCTT (R) TGGCTTTCCTGGTCCTAATCCTGA	70
*Vim*	(F) GAATGACCGCTTTGCCAACTACAT (R) GCTTCCTCTCTCTGGAGCATCTCCT	267
*Gusb*	(F) AATGAGCCTTCCTCTGCTCT (R) AACTGGCTATTCAGCTGTGG	227
*Rpl13a*	(F) ATGACAAGAAAAAGCGGATG (R) CTTTTCTGCCTGTTTCCGTA	215
*Pgk1*	(F) GCAGATTGTTTGGAATGGTC (R) TGCTCACATGGCTGACTTTA	185

### Statistical Analysis

All quantitative data is expressed as mean ± standard error. For differential expression of RNA transcripts analyzed by RNA-Seq, statistical analysis was completed using Cuffdiff and CummeRbund [53]. Statistical analysis for pathways and networks analysis was completed by IPA. Statistical analyses of quantitative data were completed using SPSS v.22.

## Results

### Control and SMA mESCs Differentiate into Motor Neurons *in vitro*

**Figure 2:**
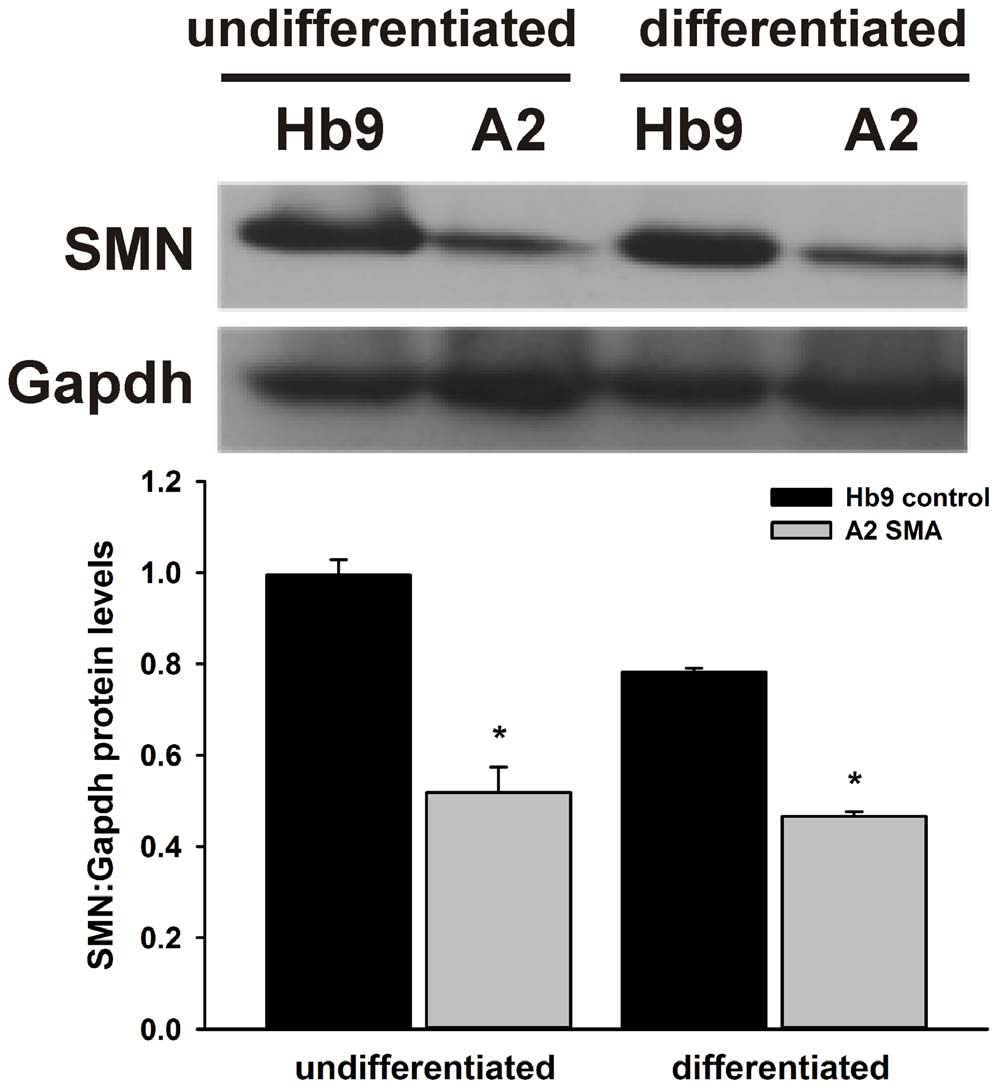
Expression of total SMN protein in undifferentiated as well as MN-differentiated control and SMA cells. Representative immunoblots of both differentiated and undifferentiated mESCs revealed a significantly reduced protein expression of SMN in A2 SMA cell lines compared to Hb9 control cells. SMN levels were normalized to Gapdh. C =  control, S =  SMA. Asterisks indicate significant differences compared to controls (*p<0.05, Student's *t* test).

Motor neurons (MNs) were generated from HB9:eGFP-expressing control (*SMN2^+/+^;mSmn^+/+^*, Hb9) and SMA (*SMN2^+/+^;mSmn^−/−^*, A2) mESCs using a combination of growth factors and morphogens [47] as outlined in **Figure 1**. We first examined the effect of MN differentiation of the levels total SMN protein levels (*mSmn* and the *SMN2* transgene) in Hb9 and A2 cells. Total SMN protein levels were indeed reduced in undifferentiated as well as in differentiated A2 cells relative to Hb9 cells (**Figure 2**). The total SMN protein levels were similar between undifferentiated and differentiated Hb9 as well as for A2 cells; the differentiation conditions thus do not affect total SMN protein expression.

**Figure 3:**
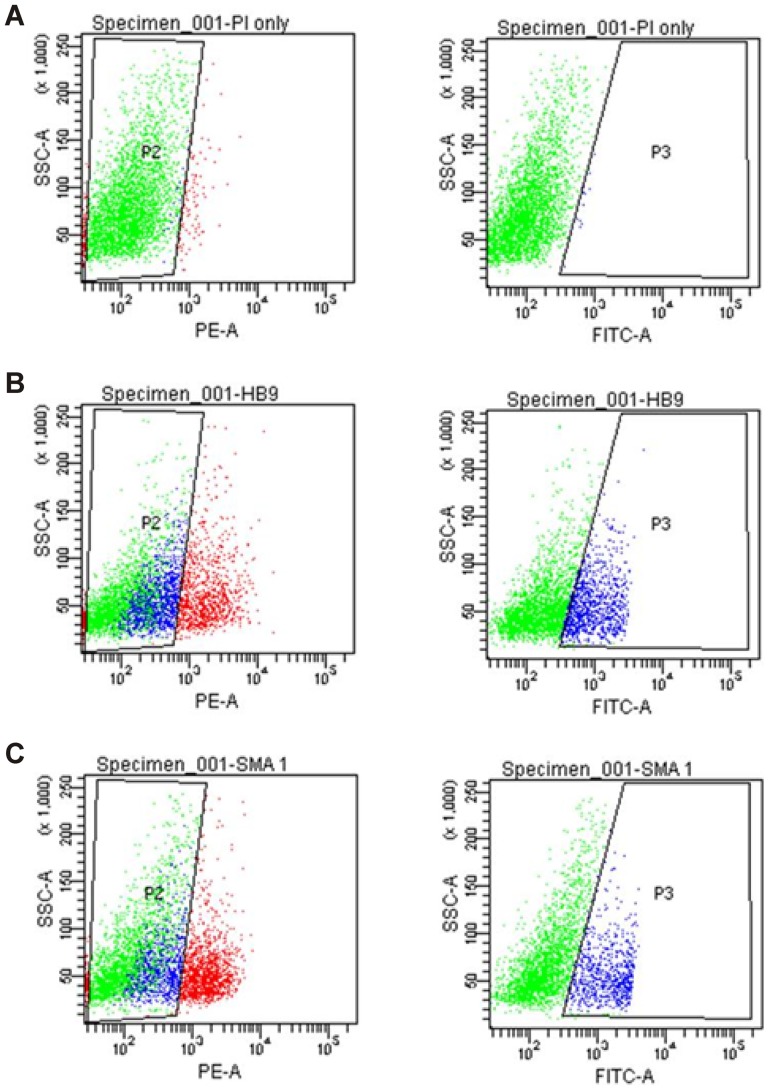
Efficiency of differentiation of Hb9 control and A2 SMA mESCs into MNs. Differentiated cells were analyzed and sorted using flow cytometry. Representative dot plots are shown. Cells treated with PI alone were used as a control (**A**). The proportion of MNs in the Hb9 control (**B**) and A2 SMA (**C**) mESCs upon differentiation is similar.

We next determined the effect of SMN deficiency on the differentiation potential of mESCs into MNs. FACS analysis showed the differentiation efficiency of viable control Hb9 cells (**Figure 3B**; 17.7±6.1%) was similar to that observed in viable A2 SMA cells (**Figure 3C**; 16.3±3.2%). Reduced expression of SMN similar to that observed in SMA does not affect the ability of mESCs to differentiate into MNs.

**Figure 4:**
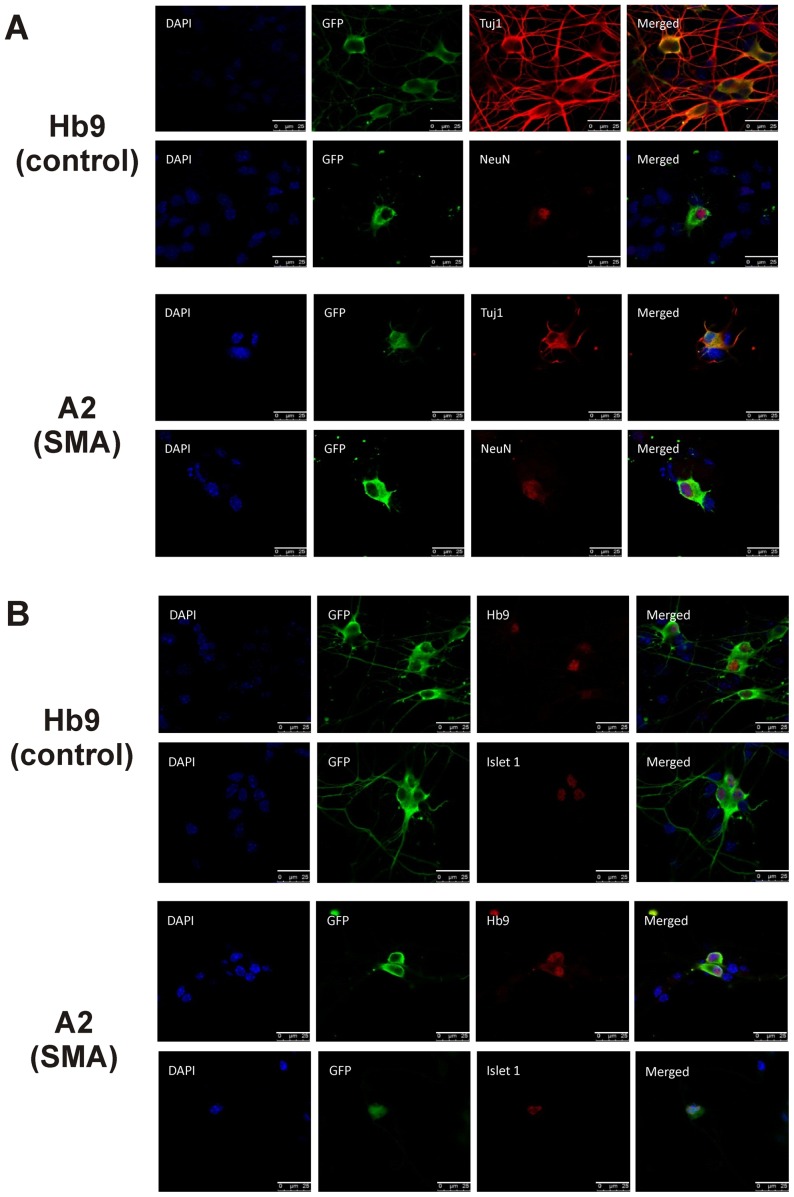
Immunofluorescent characterization of mESC derived control and SMA MNs. Hb9 control and A2 SMA mESCs were directed to differentiate into MNs, dissociated and then grown on coated coverslips in the presence of neurotrophic factors for 3 days. Cells were then immunostained with antibodies against GFP (green) and either (**A**) the panneuronal markers Tuj1 (β3-tubulin) or NeuN (Fox3) or (**B**) the MN-specific markers Hb9 and Islet 1 (red). Nuclei were counterstained with Hoescht 33342 (blue). The scale bar represents 25 µm.

To show that the differentiated cells from Hb9 and A2 mESCs were morphologically MNs, these mESCs were directed to differentiate into MNs and then plated in the presence of neurotrophic factors to induce axonal growth and arborization (**Figure 1**). The GFP+ Hb9 (control in **Figure 4**) and A2 (SMA in **Figure 4**) cells were characterized using various neuronal markers to test their MN identity. The presence of β-III tubulin (Tuj1), a neuron-specific tubulin isoform, as well as post-mitotic neuronal marker NeuN (also known as Fox-3) confirmed that the GFP+ cells were indeed neurons (**Figure 4A**). As expected, differentiated Hb9 and A2 cells expressed MN specific markers Hb-9 and Islet-1 (**Figure 4B**) [58], [59]. One noticeable difference was the number of GFP+ neuron-like cells present on coverslips after plating and growing for 3 days. Coverslips with differentiated Hb9 cells had an abundance of GFP+ cells whereas there were very few neuron-like GFP+ cells on coverslips from A2 SMA cells. This reduced viability of SMA mESC-derived MNs was consistent with a previous report [39]. Thus, both the control and SMA mESCs can be directed to differentiate into MNs and SMN-deficient SMA MNs are less viable than control MNs derived from mESCs.

### Analysis of RNA-Seq Data from Control and SMA mESC-Derived MNs

Whole transcriptome differential expression between FACS-collected Hb9 and A2 MNs was completed using massively parallel RNA sequencing (RNA-Seq). The total number of reads produced from each sample (n = 3/genotype) was around 100 million reads per sample. The quality of the trimmed reads—as assessed with FastQC—was very high in that the sequence quality had a Phred score of around 39 and an average base Phred quality score was around 32. As a point of reference, a Phred quality score of 30 corresponds to a base call accuracy of 99.9%.

**Figure 5:**
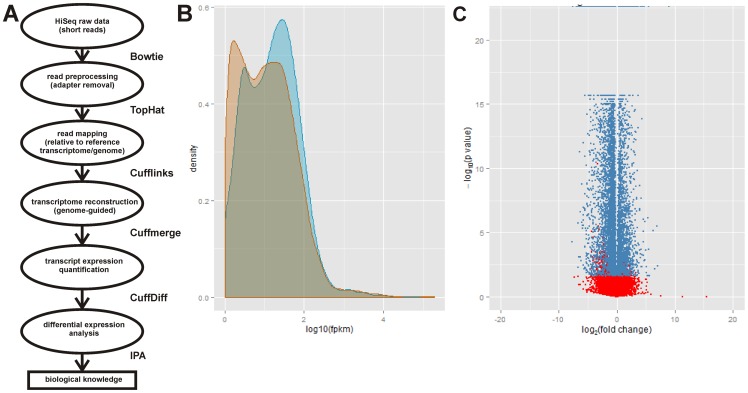
RNA-Seq-identified differentially expressed transcripts between mESC-derived Hb9 control and A2 SMA MNs. (**A**) Flowchart outlining the analysis of RNA sequencing data. The programs used for each step are shown to the right of the flowchart. (**B**) Area plot showing the distributions of FPKM values of significant transcripts in Hb9 control (blue) and A2 SMA (orange) MNs. (**C**) A volcano plot showing the log2-fold difference of statistically significant (p = 0.05) transcripts (shown in blue) between Hb9 control and A2 SMA MNs.

The trimmed sequencing reads were then mapped to the mouse reference genome (**Figure 5A**). The total number of mapped reads per sample ranged between 29,763,880 and 44,570,352 (**Table 2**); between 69% and 76% of reads were uniquely mapped to the reference genome. The difference in the average number of reads between Hb9 and A2 MNs samples was not statistically significant (p = 0.22). Transcripts were assembled and transcript abundance measured in FPKM; the expression levels for each transcript were then compared between Hb9 and A2 MNs. The distributions of transcript abundances—as measured in fragments per kilobase of exon per million fragments mapped (FPKM)—were different between A2 SMA and Hb9 control MNs (**Figure 5B**). Overall, there were 41,945 expressed transcripts in both Hb9 and A2 MNs of which 10,058 transcripts were found to be differentially expressed in Hb9 and A2 MNs (p<0.05). Out of the 10,058 statistically significant differentially expressed transcripts, 3094 were upregulated in A2 SMA samples compared to Hb9 control and 6964 were downregulated (**Figure 5C**). A list of differentially expressed RNA transcripts is provided in **Table S1**.

**Table 2:** RNA-Seq reads mapped to NCBI mouse genome build 37 using TopHat.

	Control			SMA		
	Sample 1	Sample 2	Sample 3	Sample 1	Sample 2	Sample 3
**Total Reads**	29,763,880	14,863,496	36,877,629	37,203,080	32,327,505	44,570,352
**Reads Removed**	23.86%	22.74%	23.65%	29.20%	28.81%	28.16%
**Reads Mapped**	74.49%	75.50%	74.88%	69.33%	69.67%	70.60%

Since the A2 SMA cells are homozygous for the targeted deletion of *mSmn* (*Smn1*), the levels of *Smn1* mRNA is expected to be lower in A2 SMA MNs. Indeed, the level of *Smn1* mRNA was reduced by 4.7-fold in A2 SMA MNs when compared to Hb9 controls MNs (**Table S1**). The mRNA transcript data (RNA-Seq) and the protein results (Smn immunoblot in **Figure 2**) were consistent. Interestingly, *Hb9* (*Mnx1*) transcripts levels were not significantly different between A2 and Hb9 MNs. This observation was supported by the FACS analysis in **Figure 3**.

### Pathway and Network Analysis of Differentially Expressed Transcripts in SMA ESC-derived MNs

**Figure 6:**
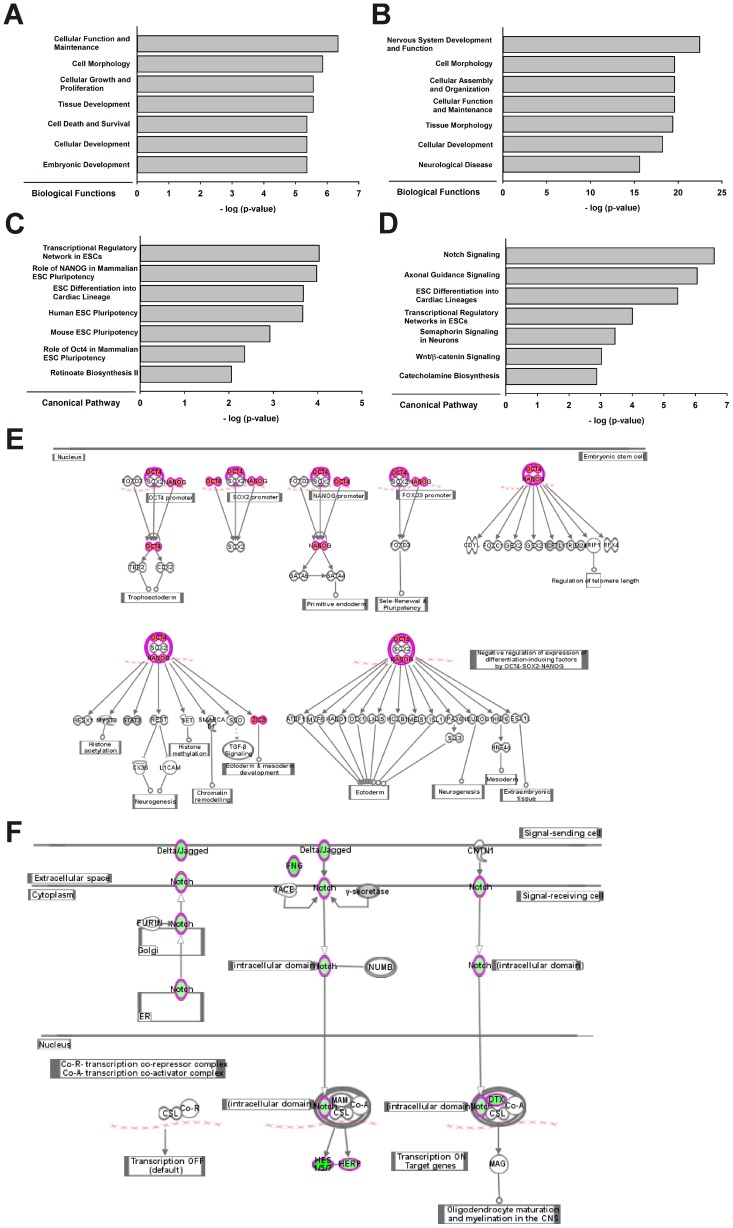
Gene network analysis of differentially expressed transcripts between mESC-derived Hb9 control and A2 SMA MNs. Ingenuity Pathways Analysis (IPA) of biological functions for transcripts (**A**) upregulated or (**B**) downregulated in A2 SMA MNs compared to Hb9 control MNs. IPA of canonical pathways for transcripts (**C**) upregulated or (**D**) downregulated in A2 SMA MNs compared to Hb9 control MNs. IPA was performed on upregulated or downregulated transcripts with at least a 2-fold change and a p value less than or equal to 0.05. The top 7 canonical pathways or biological functions were shown in the bar graphs. The top canonical pathway for (**E**) upregulated (Transcriptional Networks in ESCs) or (**F**) downregulated (Notch Signaling) transcripts in A2 SMA MNs compared to Hb9 control MNs. Downregulated transcripts are green while the upregulated transcripts are red.

Ingenuity Pathway Analysis (IPA) was used to determine the biological relevance of the differentially expressed transcripts between A2 SMA and Hb9 control mESC-derived MNs. IPA uses the Ingenuity Knowledge Base, which is a manually curated repository of literature-based biological and pharmacological data (www.ingenuity.com) [55]. 77 biological functions were overrepresented (with a Fisher's exact test p-value cutoff of 0.05) using the list of transcripts upregulated in A2 SMA MNs, the 7 highest scoring entries are shown in **Figure 6A**. Many of these highest scoring entries are related to developmental functions. In the downregulated transcripts, there were 62 overrepresented biological functions (**Figure 6B**) with many of these highest scoring functions involving nervous system development and disease.

We also used IPA to identify canonical pathways that were overrepresented in the lists of transcripts either upregulated or downregulated in A2 SMA MNs. Most of the highest scoring overrepresented canonical pathways (6 out of 14) from the upregulated transcripts were related to ESC pluripotency (**Figure 6C** with the top canonical pathway, *Transcriptional Regulatory Networks in ESCs*, shown in detail in **Figure 6E**). Many of the highest scoring canonical pathways identified from the list of downregulated transcripts (5 out of 19) were involved with nervous system development (**Figure 6D**). In fact, the top downregulated canonical pathway is *Notch signaling* (shown in detail in **Figure 6F**).

**Figure 7:**
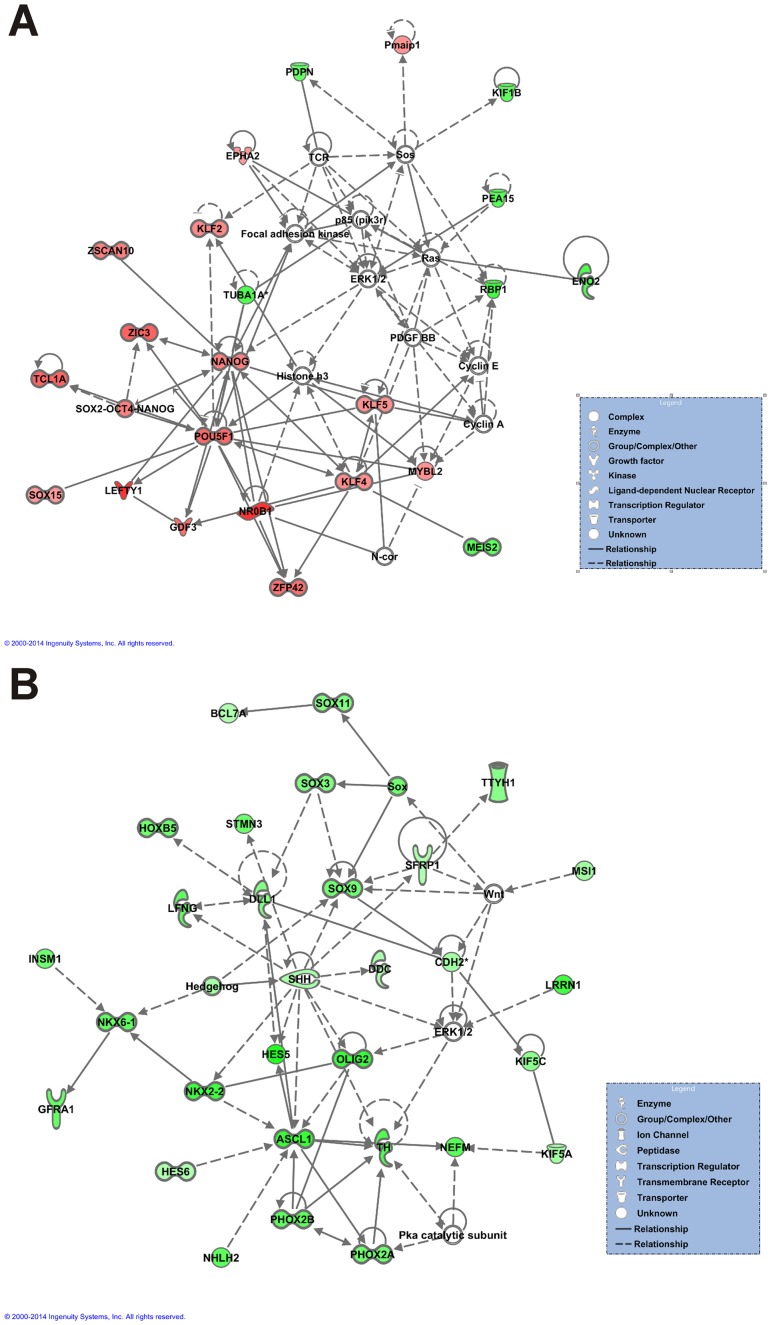
Functionally regulated gene networks in mESC-derived A2 SMA and Hb9 control MNs. The highest scoring interactomes generated by IPA from the (A) upregulated or (**B**) downregulated transcripts in A2 SMA MNs when compared against Hb9 control MNs. Each node represents a gene product in the network. Upregulated gene products are shown in red while the downregulated gene products are shown in green; the intensity of color indicates the degree of regulation. The uncolored nodes represent unregulated gene products which have a relationship with a colored node based on the literature. A line between two nodes represents a biological relationship with the line length indicating the amount of literature-based evidence supporting this relationship. The solid lines represent direct interactions while the dashed lines represent indirect interactions.

In addition to identify biological functions and canonical pathways altered as a result of SMN deficiency in MNs, IPA can also integrate these functions and pathways to identify functionally interacting gene networks, or interactomes. We analyzed our lists of transcripts upregulated or downregulated in A2 SMA MNs for overrepresented interactomes. IPA network scores greater than or equal to 15 were considered significant [54]. Using these criteria, there were 2 significant interactomes identified from analysis of the upregulated transcripts and 13 from analysis of the downregulated transcripts. The top upregulated interactome (score of 59; **Figure 7A**) contained many gene products involved in ESC pluripotency and cellular development, growth and proliferation. Gene products with roles in nervous system development and function were present in the top scoring interactome of the downregulated transcripts (score of 60; **Figure 7B**). Taken together, a systems biology-based comparison of A2 SMA and Hb9 control mESC-derived MNs shows that functions, pathways and gene networks involved in pluripotency are upregulated in SMA MNs while those involved in nervous system development and function are downregulated in SMA MNs.

### *In Silico* Identification of Upstream Regulators in SMA mESC-derived MNs

**Figure 8:**
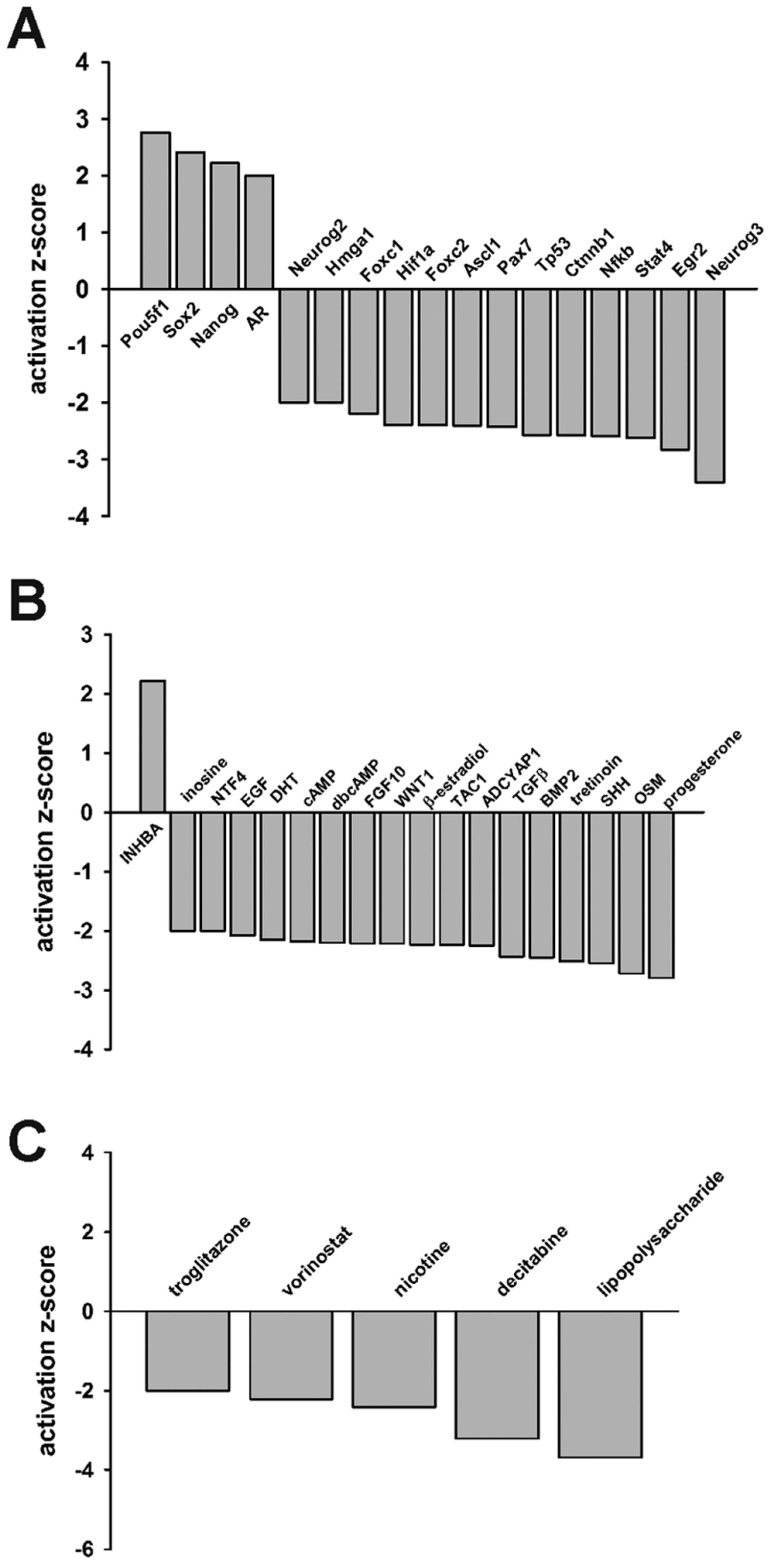
Upstream regulator analysis of differentially expressed transcripts between mESC-derived Hb9 control and A2 SMA MNs. IPA of significantly modified upstream (**A**) transcriptional regulator, (**B**) endogenous signaling and (**C**) drug pathways in upregulated or downregulated transcripts in A2 SMA MNs compared to Hb9 control MNs. Significant upstream regulators were identified as those having an activation z-score greater than or equal to 2.0 for activated regulators or less than or equal to −2.0 for inhibited regulators.

Using the Upstream Regulator Analysis (URA) component of IPA, we can identify possible upstream regulatory molecules that may be affecting changes in gene expression [55]. A list of such upstream regulators was compiled by URA using the transcripts which were upregulated or downregulated in A2 SMA mESC-derived MNs. We divided these upstream regulators into 3 groups: transcriptional regulators, signaling components and drugs. As shown in **Figure 8A**, 4 transcriptional regulators were activated in SMA MNs while 13 transcriptional regulators were inhibited in SMA MNs. Of these 17 transcriptional regulators identified, the transcript levels of only three—*Pou5f1* (*Oct-4*), *Nanog* and *Ascl1*—were significant altered in A2 SMA MNs (3.0-fold increase, 2.5-fold increase and 5.6-fold decrease, respectively).

In addition to transcriptional upstream regulators, the intracellular effects of 18 signaling molecules were identified as being significantly modified in A2 SMA MNs (**Figure 8B**). These signaling molecules include growth factors and cytokines like transforming growth factor β (TGFβ) and neurotrophin-4 (NTF-4), intracellular second messengers like cyclic AMP (cAMP) as well as nuclear receptor ligands such as dihydrotesterone (DHT) and tretinoin (RA). The effects of all of these signaling molecules except for inhibin βA (INHBA) were attenuated in A2 SMA MNs. The levels of *Sonic hedgehog* (*Shh*) mRNA were significantly reduced (2.4-fold) in A2 SMA MNs relative to Hb9 control MNs.

URA also predicted that the effects of 5 drugs would be inhibited in A2 SMA MNs as compared to Hb9 control MNs (**Figure 8C**). The drugs identified were troglitazone (a PPARγ agonist), vorinostat (suberoylanilide hydroxamic acid (SAHA), a histone deacetylase inhibitor), nicotine (acetylcholine receptor agonist), decitabine (5′-aza-2′-deoxycytidine, a DNA methyltransferase inhibitor) and the inflammatory mediator lipopolysaccharide (LPS).

### Validation of Differentially Expressed Transcripts Identified by RNA-Seq

As a first step toward the validation of the RNA-Seq data, we needed to reduce the number of differentially expressed transcripts to a more manageable list. The initial list of differentially expressed transcripts (using a p≤0.05 as a criterion) was re-analyzed using more stringent criteria. By filtering these data for differentially expressed transcripts with a p-value less than or equal to 0.01 and a greater than 4-fold change, the list of candidate transcripts was reduced to 286 upregulated transcripts and 814 downregulated genes. The filtering of these data was further refined so as to include only those transcripts with a FPKM >30 (**Figure 5B**). With this added refinement, there were 27 transcripts upregulated in A2 SMA MNs and 220 downregulated transcripts.

**Figure 9:**
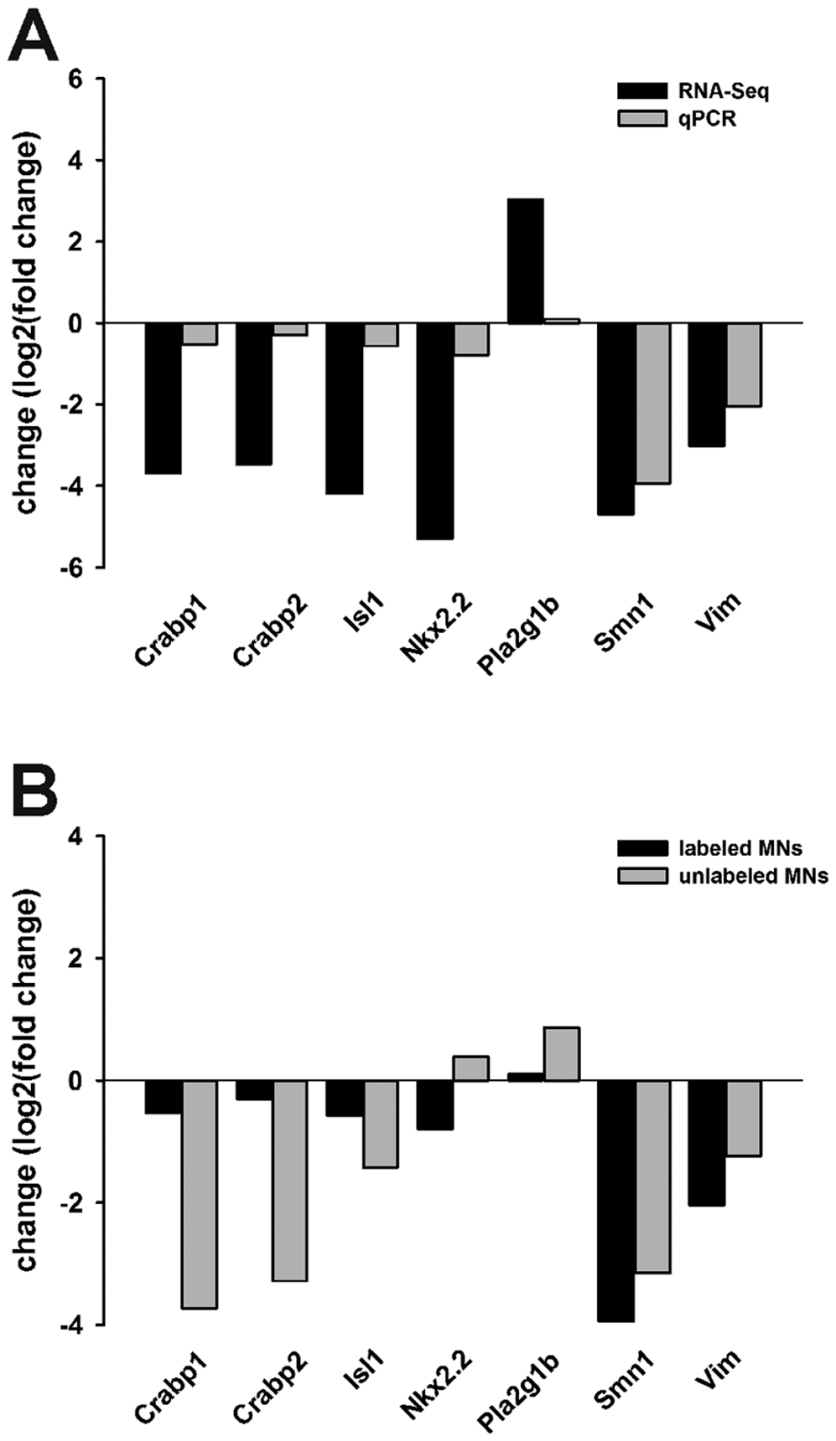
Validation of differentially expressed transcripts determined by RNA-Seq using qRT-PCR. The levels of *Crabp1*, *Crabp2*, *Isl1*, *Nkx2.2*, *Pla2g1b*, *Smn1* and *Vim* transcripts were measured in total RNAs from control and SMA mESC-derived MNs. (**A**) The magnitude of change (log_2_(fold change)) of selected transcripts in A2 SMA MNs relative to Hb9 control MNs (n = 3/genotype) as determined by RNA-Seq (black bars) or qRT-PCR (grey bars). (**B**) The magnitude of change of selected transcripts in HB9:eGFP-labeled (black bars) or unlabeled (grey bars) SMA MNs (A2 and E2 cells, respectively) relative to control MNs (Hb9 and C4 cells, respectively; n = 3/genotype).

To validate the results from the analysis of the RNA-Seq data, we measured changes in the levels of selected differentially expressed transcripts between Hb9 and A2 MNs by qRT-PCR. We selected *Smn1* since this gene is knocked out in SMA A2 cells. In addition, six other transcripts were selected based on their strong changes in transcript levels as shown by RNA-Seq: *cellular retinoic acid binding protein 1* (*Crabp1*), *Crabp2*, *Islet-1* (*Isl1*), *NK2 homeobox 2* (*Nkx2.2*), *phospholipase A2, group 1B* (*Pla2g1b*) and *vimentin* (*Vim*). The sample RNAs used for qRT-PCR (n = 3/genotype) were not the same as those used for RNA-Seq so they represent biological replicates as opposed to technical replicates [60]. The differences in transcript levels between Hb9 and A2 MNs determined by qRT-PCR followed the same trends as those determined by RNA-Seq although the magnitudes of change were generally higher in the RNA-Seq data (**Figure 9A**). RNA-Seq is more sensitive than qRT-PCR at detecting changes in transcript levels.

We next determined if the changes in RNA levels observed in these SMA mESC-derived MNs were unique to these specific cells. Control and severe SMA mESCs that do not contain the HB9-eGFP reporter transgene—C4 and E2 cells, respectively—[46] were directed to differentiate into MNs. The extracted total RNAs from C4 and E2 MNs were analyzed by qRT-PCR. As shown in **Figure 9B**, with the exception of *Nkx2.2*, the changes in transcript levels between C4 and E2 MNs were similar to those observed between eGFP-labeled Hb9 and A2 MNs. One possible explanation for this discrepancy in changes in *Nkx2.2* levels is that the MNs were not separated from other cell types after differentiation of C4 and E2 mESCs.

Using two-dimensional differential in-gel electrophoresis (2D-DIGE) and mass spectrometry, we previously identified a set of proteins that are differentially expressed between control and severe SMA mESC-derived MNs [46]. We compared this list of differentially expressed proteins with our list of differentially expressed mRNA transcripts. Of the 12 proteins identified, lactate dehydrogenase B (Ldhb) and aldehyde dehydrogenase (Aldh5a1) showed corresponding changes in mRNA levels between control and SMA mESC-derived MNs (**Table 3**). 5 proteins that showed significant difference in protein levels between control and SMA mESC-derived MNs—brain creatine kinase (Ckb), tropomyosin 3 (Tpm3), ubiquitin C-terminal hydroxylase L1 (Uchl1), 14-3-3γ (Ywhag) and heat shock protein 90β (Hsp90b1)—did not exhibit any corresponding changes in mRNA levels. While some genes have similar changes in mRNA and protein expression in SMA MNs relative to control MNs, there are other genes which are differentially expressed at the protein level but not at the mRNA level. These observations suggest that differential gene regulation occurs at many levels—i.e. transcriptional vs. post-transcriptional—between SMA and control MNs.

**Table 3:** Relationship between changes in mRNA levels measured by RNA-Seq and protein levels measured by 2D-DIGE or immunoblot (*) of selected genes in normal versus SMA mESC-derived motor neurons.

Gene Symbol	Protein Name	mRNA Fold Change	Protein Fold Change
***Upregulated proteins***			
*Cdkn1a*	p21	−0.764	+41.3*
*Ldhb*	lactate dehydrogenase B	+1.08	+3.60
*Ckb*	brain creatine kinase	N.S.	+1.80
*Glo1*	glyoxalase 1	−0.970	+1.75
*Tpm3*	tropomyosin 3	N.S.	+1.75
*Anxa5*	annexin A5	−0.487	+1.70
*Uchl1*	ubiquitin C-terminal hydroxylase L1	N.S.	+1.70
*Tuba1a*	α-tubulin	−2.37	+1.50
***Downregulated proteins***			
*Aldh5a1*	aldehyde dehydrogenase	−0.952	−1.70
*Ywhag*	14-3-3γ	N.S.	−1.70
*Hsp90b1*	Heat shock protein 90β	N.S.	−1.80
*Hspa9*	Heat shock protein 70	+0.812	−2.20

The protein expression data is taken from [46].

### Differential Expression of Validated Transcripts in SMA Mice

**Figure 10:**
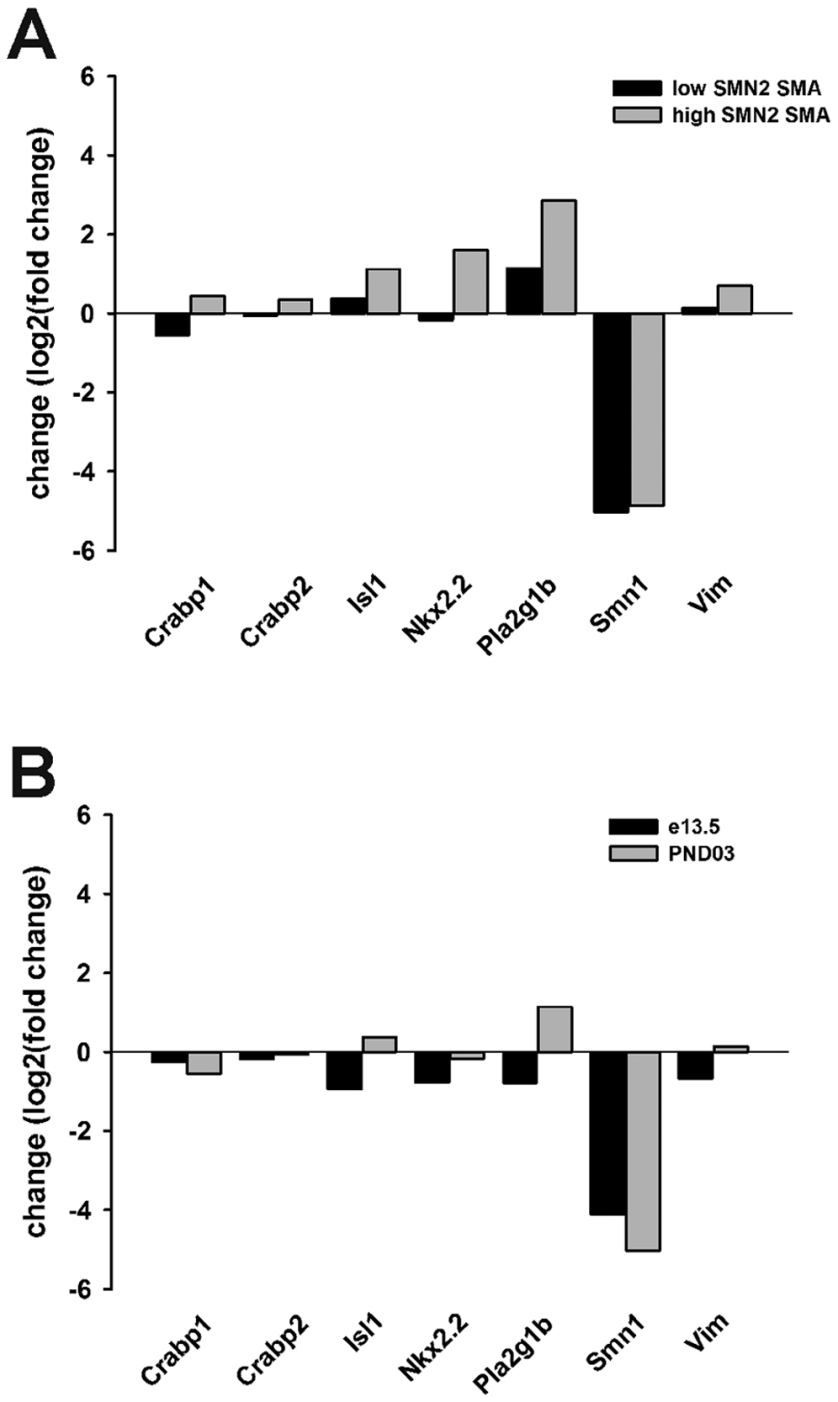
Expression of RNA-Seq-identified differentially expressed transcripts in SMA mouse spinal cords. The levels of *Crabp1*, *Crabp2*, *Isl1*, *Nkx2.2*, *Pla2g1b*, *Smn1* and *Vim* transcripts were measured in spinal cord total RNAs from control and SMA mice. (**A**) The magnitude of change (log_2_(fold change)) of selected transcripts in low copy *SMN2* SMA (*SMN2(89)^+/+^;mSmn^−/−^*; black bars) or high copy *SMN2* rescue (*SMN2(566)^+/+^;mSmn^−/−^*; grey bars) mouse spinal cord samples (n = 3/genotype) relative to control samples. (**B**) The magnitude of change of selected transcripts in low copy SMN2 SMA mouse spinal cord samples relative to controls at embryonic day 13.5 (e13.5; black bars) and postnatal day 3 (PND03; grey bars).

The levels of *Smn1*, *Crabp1*, *Crabp2*, *Isl1*, *Nkx2.2*, *Pla2g1b* and *Vim* transcripts were examined in total RNA samples from control (*SMN2^+/+^;mSmn^+/+^*) and severe SMA (*SMN2^+/+^;mSmn^−/−^*) mouse spinal cords in order to determine if the changes observed in mESC-derived MNs could also be observed *in vivo*. Mouse embryos of similar genotypes were used to generate the mESCs used in this study. Spinal cord total RNAs (n = 3/genotype) were extracted from PND03 mice; severe SMA mice at this time point begin to show signs of motor dysfunction [34]. Similar to SMA mESC-derived MNs in culture, *Smn1*, *Crabp1*, *Crabp2* and *Nkx2.2* transcript levels were reduced while *Pla2g1b* levels were increased in SMA spinal cords (**Figure 10A**). Surprisingly, *Isl1* and *Vim* mRNA levels were elevated in SMA spinal cords at PND03 even though these transcripts were reduced in SMA MNs. The samples isolated from SMA mouse spinal cords contain RNAs from many different types of neurons aside from MNs as well as other cell types such as astrocytes and oligodendrocytes. This sample heterogeneity could explain the discrepancies observed between mESC-derived SMA MNs and SMA spinal cords.

Increasing *SMN2* copy numbers can improve the phenotype and survival of severe SMA mice [34], [36]. In fact, *SMN2* transgenic SMA mice with 8 (*SMN2(566)^+/−^;mSmn^−/−^*) or 16 copies (*SMN2(566)^+/+^;mSmn^−/−^*) of the transgene display no motor phenotype [34]; in other words, the SMA phenotype is rescued. When comparing relative changes in *Smn1*, *Crabp1*, *Crabp2*, *Isl1*, *Nkx2.2*, *Pla2g1b* and *Vim* transcript levels in low-copy *SMN2* SMA mice with those observed in high-copy *SMN2* rescue mice at PND03, the relative changes in each of these transcripts—except for *Smn1*—were increased in high-copy rescue mice as opposed to relative changes in low-copy *SMN2* SMA mice (**Figure 10A**). *Smn1* mRNA levels were still reduced in high-copy *SMN2* rescue mice since these mice are still nullizygous for *mSmn*.

We also wanted to determine if the changes observed in severe SMA mouse spinal cords are regulated developmentally. The levels of *Smn1*, *Crabp1*, *Crabp2*, *Isl1*, *Nkx2.2*, *Pla2g1b* and *Vim* transcripts were measured in severe SMA mice at embryonic day 13.5 (e13.5) relative to age-matched control littermates. There are no overt changes in physical characteristics or in motor neuron growth in SMA mice at this developmental age [45]. *Smn1* mRNA levels were marked reduced in the embryonic SMA mouse spinal cord as they were at PND03 (**Figure 10B**). Some transcripts, like *Isl1*, *Pla2g1b* and *Vim*, showed reduced mRNA levels at e13.5 even though the levels of these transcripts were increased at PND03. The reductions in *Crabp2* and *Nkx2.2* transcripts were more pronounced at e13.5 than at PND03. These results suggest that the changes in RNA expression of some transcripts may be developmentally regulated in SMA.

## Discussion

In this study, we compared the RNA expression profiles (transcriptomes) between SMA and normal MNs in order to identify the molecular consequences of SMN deficiency in these cells. This study is the first to examine the transcriptome profiles of isolated MNs derived from severe SMA and normal mESCs. Differential gene expression studies using cDNA microarrays have also been previously completed using primary MN cultures from *mSmn^+/−^* mice—a supposed model for mild SMA [61]—and whole spinal cords from the SMA mouse models [40]–[42]. As mentioned earlier, analysis of transcriptome profiling using RNA-Seq is advantageous because of the high signal-to-noise ratio, independence from hybridization efficiency between probes and targets and identification of novel RNA transcripts. In addition to mouse models for SMA, differential transcriptome expression analyses have been completed on other animal models of Smn deficiency [62], [63]. Recently, Zhang et al. [64] used RNA-Seq to identify differentially expressed mRNAs in PND01 SMNΔ7 SMA mouse MNs isolated by laser capture microdissection (LCM). Our study examines the transcriptome in purified, cultured MNs derived from control and severe SMA mESCs. Using this model, we can examine changes in RNA expression in a nearly homogeneous population of cells which is not possible with LCM.An advantage of using MNs derived from mESCs to compare the transcript profiles of SMA MNs to normal MNs is that large numbers of cells can be directed to differentiate into MNs. This scalability can overcome two obstacles of using primary MN cultures from SMA mouse spinal cords, low yield of MNs from SMA mouse embryos and the fragility of these affected MNs. Numerous studies in mice and zebrafish have shown that SMA MNs are affected in a cell autonomous manner [26], [65]–[67] which would suggest that examining the transcriptome profiles of isolated SMA MNs could provide relevant information on the pathobiology of this disease. In isolated mESC-derived SMA MNs, the transcriptomes will not show the effects of target (i.e. skeletal muscle) innervation and trophic signaling from skeletal muscle, sensory neurons or glia. Future experiments will compare the transcriptomes of mESC-derived SMA MNs grown in isolation with those grown in the presence of myotubes and/or glial cells so as to determine those gene regulatory events which are intrinsic to SMA MNs (i.e. those grown in isolation) and those which are dependent on environmental cues (i.e. those grown in the presence of target or support cells).

MNs are the primary cells affected by reduced SMN expression in SMA. Ectopic overexpression of SMN in the neurons of severe SMA mice rescues the primary disease phenotype in these mice while transgenic overexpression of SMN in mature skeletal muscle does not improve the SMA phenotype [68]. Conditional expression of SMN in the developing MNs of SMA mice—using either the Hb9 or Olig2 promoters as drivers—significantly ameliorates the SMA phenotype [65]–[67]. Martinez *et al.* [66] also show that conditional expression of SMN in SMA skeletal muscle may help grow and maintain muscle independent of MNs. Increasing SMN expression outside of the nervous system with either splice-switching oligonucleotides [69] or adeno-associated virus (AAV) vectors [70], [71] markedly improves the phenotype and survival of SMA mice. These studies suggest that comparative analysis of SMA MN transcriptomes from these models may provide limited insight into the pathobiology of SMA; however, it is appropriate to examine the transcript profiles of isolated SMA MNs since they are affected in a cell autonomous fashion [26], [65]–[67].

The copy number of *SMN2* modifies the severity of the SMA in humans [13]–[20]. *SMN2* also acts as a phenotypic modifier in transgenic mouse models for SMA [34]–[36]. Increasing SMN expression in MNs *in vivo* by pharmacological induction of *SMN2* expression [72]–[74] or SMN gene replacement therapies [70], [71], [75]–[77] improves the phenotype and survival of SMA mice. The levels of certain mRNA transcripts such as *Crabp1*, *Crabp2* and *Nkx2.2* were elevated in high copy *SMN2* rescue mice even though the levels of these transcripts were reduced in low copy *SMN2* severe SMA mice (**Figure 10A**). Increasing SMN2 expression rescues molecular phenotype of Smn-deficient MNs *in vivo*.

Many of the biological pathways and networks that were overrepresented in those transcripts upregulated in A2 SMA MNs involved ESC pluripotency (**Figures 6A and 6C**). The transcription factors Nanog, Pou5f1 (Oct4), and Sox2 are considered to be hallmarks of ESC pluripotency [78]. mRNA transcripts for all three of these factors were upregulated in SMA mESC-derived MNs (**Figure 6E**). UPA of the differentially expressed transcripts revealed that these three pluripotency transcription factors were activated in A2 SMA mESC-derived MNs (**Figure 8A**). Several gene products work with these three transcription factors to regulate pluripotency in ESCs. Klf2 regulates the expression of *Sox2* [79]. *Klf2* transcript levels were increased in SMA mESC-derived MNs by 2.3-fold (**Table S1**). *Zic3*—whose transcript levels were increased 3.1-fold in SMA mESC-derived MNs—is directly regulated by all three transcription factors [80]. Zscan10 (also known as Zfp206), whose mRNA levels are elevated by 2.5-fold in SMA mESC-derived MNs (**Table S1**), helps maintain pluripotency by jointly functioning with Sox2 and Oct4 [81]. In SMA mESC-derived MNs, the pluripotency marker *Dppa5* (also known as *ESG-1* [82]) transcript levels were increased by 1.2 fold (**Table S1**). The levels of *Dppa5* mRNA are significantly upregulated—as shown with microarray analysis—in the pre-symptomatic severe SMA mouse spinal cord [42]. Two genes that are also implicated in the maintenance of pluripotency—*Mybl2* and *Zfp42* (*Rex1*) [83], [84]—were upregulated by 2.2- and 2.8-fold, respectively, in SMA mESC-derived MNs (**Table S1**).

Neuronal development and activity functions are impaired in A2 SMA mESC-derived MNs given that these biological pathways and networks were overrepresented in the downregulated RNA transcripts (**Figures 6B and 6D**). Vimentin (Vim) is an intermediate filament protein that is transiently expressed by all neuronal precursors. It may have a role in neurite outgrowth in developing hippocampal neurons [85]. Nkx2.2 is homeobox transcription factor which is activated downstream of Shh; it has a primary role in ventral neuronal patterning and in generating interneurons [86]. Islet1 (Isl1) and Olig2 are transcription factors expressed during and are necessary for MN development [37]. *Vim*, *Nkx2.2* and *Isl1* mRNA levels were markedly reduced in mESC-derived SMA MNs (**Figure 9**) and in e13.5 SMA mouse spinal cords (**Figure 10**); *Olig2* mRNA levels were reduced by 5.5-fold in A2 SMA mESC-derived MNs (**Table S1**). Using microarray analysis, the expression of *neurexin2a* (*nrxn2a*) is downregulated in zebrafish embryos with reduced *smn* expression [63]. *Nrxn2a* transcript levels are also reduced in the spinal cord of severe SMA mice [63]. Nrxn2 deficiency has been implicated in altered neuronal excitability. We found that *Nrxn2a* mRNA levels were reduced by 2.9-fold in SMA mESC-derived MNs (**Table S1**).

The Notch pathway, via cell-cell interactions, stimulates neural precursors cells to differentiate into neurons and glial cells [87]. Notch pathway signaling was the top canonical pathway overrepresented in the list of transcripts downregulated in A2 SMA mESC-derived MNs (**Figure 6D**). Many of the components of this pathway including Notch as well as its extracellular ligands Jagged and Delta were reduced in A2 SMA mESC-derived MNs (**Figure 6F**). *Neurogenin-3* (*Neurog3*) mRNA levels were reduced by 1.3-fold in SMA mESC-derived MNs (**Table S1**) and Neurog3 was identified as one of the inhibited transcription factor activities in the UPA (**Figure 8A**). These results are surprising given that Notch protein immunolocalization is increased on the ventral horn motor neurons of SMNΔ7 SMA mice at PND11 [88]. Jagged1 and Delta1 protein immunolocalization is also increased on adjoining astrocytes in these mice. The differences between our observations and those of Carabello-Miralles *et al.* [88] could be the result of different SMA models being used (low copy SMN2 severe SMA vs. SMNΔ7 SMA) or types of gene regulation being examined (transcription vs. translation). Cell-cell interactions between neurons and glia that regulate the expression of Notch signaling ligands and receptors may not be apparent in our model system (purified MNs in culture); however, both studies document reduced Neurog3 expression and activity in SMA MNs ([88] and *this work*).

RA is known for driving cellular differentiation and has a neuralizing effect during neuronal development. UPA of the differentially expressed transcripts in A2 SMA mESC-derived MNs showed that all trans-RA (tretinoin) signaling was inhibited in SMA MNs (**Figure 8B**). Transcripts whose protein products are involved in RA signaling and metabolism were downregulated in A2 SMA mESC-derived MNs. Cellular retinol-binding protein (Rbp1) and the cellular RA binding proteins Crabp1 and Crabp2 are expressed in early mouse embryos and may play a role in the development of the CNS [89], [90]. The expression of the pluripotency-related protein Zfp42 (Rex1)—whose mRNA was upregulated in A2 SMA mESC-derived MNs, as mentioned earlier—is repressed by RA in F9 teratocarcinoma cells [91]. *Rbp1*, *Crabp1* and *Crabp2* mRNA levels were reduced in SMA mESC-derived MNs (**Figure 9** and **Table S1**) as well as in severe SMA spinal cords (**Figure 10**). Microarray analysis of PND05 (late-symptomatic) severe SMA spinal cord mRNAs also identify impairments in RA signaling and metabolism [42]. RA regulates many phases during MN differentiation [92]. RA has been implicated in its ability to induce neurogenesis by blocking fibroblast growth factor (FGF) signaling [93]. Furthermore, RA and FGF signaling are sufficient to induce MN differentiation independent of Shh signaling [94].

The upregulation of pluripotency-related transcripts and the downregulation of transcripts related to neuronal development and activity identified in this study suggest that SMA mESCs may not be differentiating into MNs as efficiently as normal mESCs. The difference between the number of MNs differentiated from A2 SMA and Hb9 control mESCs was not significant (**Figure 3**). This observation was based on eGFP expression that was driven by the MN promoter HB9. HB9 is a late-stage MN marker [37]. Consistent with the FACS analysis, *Hb9* mRNA expression was not significantly altered in SMA mESC-derived MNs even though the mRNA levels for early-stage MN markers like *Isl1* and *Olig2* were reduced in A2 SMA mESC-derived MNs. The levels of proteins found in MNs—like choline acetyltransferase (ChAT), HB9 and neurofilament—are not altered by SMN deficiency [46]. Taken together, our observations suggest that SMA MNs can initially develop normally but they do exhibit changes in transcripts related to pluripotency and neural development. These transcripts may have novel functions in MNs aside from development.

Microarray analysis of differential gene expression between control and severe SMA spinal cord transcript pools suggest that SMA is a neurodegenerative disease instead of a neurodevelopmental disorder [42]. We did not observe an overrepresentation of apoptosis and cell death transcripts in the pathway and network analyses of our differentially expressed transcriptome data. There is no noticeable cell death in the ventral horn of the spinal cord of severe SMA mouse embryos even though cell death can be detected in the telencephalon [95]. When A2 SMA mESC-derived MNs were plated onto coverslips, we did observe a time-dependent loss of cell viability and reduced neurite outgrowth (**Figure 4**). Similar reduced neurite outgrowth and cell death are observed in MNs differentiated from induced pluripotent stem cell (iPSC) lines derived from human SMA fibroblasts [96]–[99]. Primary MNs cultured from severe SMA mouse embryos exhibit reduced neurite lengths [30]. Knockdown of *Smn* in zebrafish embryos with morpholino antisense oligonucleotides results in defects in motor axons suggesting early developmental defects [26]. We found that many of the biological pathways downregulated in A2 SMA mESC-derived MNs involved axonal guidance (**Figure 6D**). No developmental defects in motor axon formation occur in severe SMA mice [26], [95]. In both mouse and fruit fly models for SMA, the maturation and maintenance of neuromuscular junctions is defective and this defect may be the result of denervation injury and/or neurodevelopmental delay [45], [100]–[103]. Comparison of presymptomatic control and SMNΔ7 SMA MNs using RNA-Seq reveal deficits in transcripts related to synaptogenesis including agrin (*Agrn*) [64]. In our isolated mESC-derived SMA MNs, we also observed a significant (0.85-fold) decrease in Agrn mRNA levels (**Table S1**). Our transcriptome analysis suggests that there may be neurodevelopmental delays in SMA MNs; however, SMA MNs develop normally initially and form connections with target muscles but these connections then atrophy for unknown reasons (reviewed in [21]). Upregulation of pluripotency and cell proliferation transcripts as well downregulation of neuronal development-related transcripts in SMA MNs may be a consequence of denervation and axonal degeneration.

In conclusion, we have identified distinct gene expression patterns in SMA MNs when compared to normal MNs. Pathways upregulated in SMA mESC-derived MNs were involved in pluripotency and cell proliferation whereas common pathways found in the downregulated genes have shown decreases in neuronal markers commonly found in mature and developing neurons. It remains to be determined whether these neuronal marker deficits are a contributing cause or a consequence of the disease. The mechanisms underlying these changes in the transcriptome of SMA MNs will need to be examined in more detail for future studies. Comparison of SMA MN transcriptomes against normal MN RNA transcript profiles will also lead to the identification of novel targets for the development of therapeutics for SMA.

## Supporting Information

Table S1 **List of all of the annotated RNA transcripts that are differentially expressed in A2 SMA mESC-derived MNs relative to Hb9 control mESC-derived MNs.**
(XLS)

## References

[1] Crawford TO, Pardo CA (1996) The neurobiology of childhood spinal muscular atrophy. Neurobiol Dis 3: 97–110. 9173917 10.1006/nbdi.1996.0010

[2] Cuscó I, Barceló MJ, Soler C, Parra J, Baiget M, et al (2002) Prenatal diagnosis for risk of spinal muscular atrophy. Br J Obstet Gynaecol 109: 1244–1249. 12452462

[3] Pearn J (1978) Incidence, prevalence and gene frequency studies of chronic childhood spinal muscular atrophy. J Med Genet 15: 409–413. 745211 10.1136/jmg.15.6.409 PMC1013753

[4] Ben-Shachar S, Orr-Urtreger A, Bardugo E, Shomrat R, Yaron Y (2011) Large-scale population screening for spinal muscular atrophy: clinical implications. Genet Med 13: 110–114. 21233719 10.1097/GIM.0b013e3182017c05

[5] Sugarman EA, Nagan N, Zhu H, Akmaev VR, Zhou Z, et al (2012) Pan-ethnic carrier screening and prenatal diagnosis for spinal muscular atrophy: clinical laboratory analysis of >72400 specimens. Eur J Hum Genet 20: 27–32. 21811307 10.1038/ejhg.2011.134 PMC3234503

[6] Su YN, Hung CC, Lin SY, Chen FY, Chern JPS, et al (2011) Carrier screening for spinal muscular atrophy (SMA) in 107,611 pregnant women during the period 2005–2009: a prospective population-based cohort study. PLoS ONE 6: e17067. 21364876 10.1371/journal.pone.0017067 PMC3045421

[7] Lyahyai J, Sbiti A, Barkat A, Ratbi I, Sefiani A (2012) Spinal muscular atrophy carrier frequency and estimated prevalence of the disease in Moroccan newborns. Genet Test Mol Biomarkers 16: 215–218. 21950724 10.1089/gtmb.2011.0149

[8] Lefebvre S, Bürglen L, Reboullet S, Clermont O, Burlet P, et al (1995) Identification and characterization of a spinal muscular atrophy-determining gene. Cell 80: 155–165. 7813012 10.1016/0092-8674(95)90460-3

[9] Lorson CL, Hahnen E, Androphy EJ, Wirth B (1999) A single nucleotide in the *SMN* gene regulates splicing an is responsible for spinal muscular atrophy. Proc Natl Acad Sci U S A 96: 6307–6311. 10339583 10.1073/pnas.96.11.6307 PMC26877

[10] Monani UR, Lorson CL, Parsons DW, Prior TW, Androphy EJ, et al (1999) A single nucleotide difference that alters splicing patterns distinguishes the SMA gene *SMN1* from the copy gene *SMN2* . Hum Mol Genet 8: 1177–1183. 10369862 10.1093/hmg/8.7.1177

[11] Lorson CL, Androphy EJ (2000) An exonic enhancer is required for inclusion of an essential exon in the SMA-determining gene SMN. Hum Mol Genet 9: 259–265. 10607836 10.1093/hmg/9.2.259

[12] Cho S, Dreyfuss G (2010) A degron created by SMN2 exon 7 skipping is a principal contributor to spinal muscular atrophy severity. Genes Dev 24: 438–442. 20194437 10.1101/gad.1884910 PMC2827839

[13] Coovert DD, Le TT, McAndrew PE, Strasswimmer J, Crawford TO, et al (1997) The survival motor neuron protein in spinal muscular atrophy. Hum Mol Genet 6: 1205–1214. 9259265 10.1093/hmg/6.8.1205

[14] Lefebvre S, Burlet P, Liu Q, Bertrandy S, Clermont O, et al (1997) Correlation between severity and SMN protein level in spinal muscular atrophy. Nat Genet 16: 265–269. 9207792 10.1038/ng0797-265

[15] McAndrew PE, Parsons DW, Simard LR, Rochette C, Ray PN, et al (1997) Identification of proximal spinal muscular atrophy carriers and patients by analysis of SMN^T^ and SMN^C^ gene copy number. Am J Hum Genet 60: 1411–1422. 9199562 10.1086/515465 PMC1716150

[16] Prior TW, Swoboda KJ, Scott HD, Hejmanowski AQ (2005) Homozygous *SMN1* deletions in unaffected family members and modification of the phenotype by *SMN2* . Am J Med Genet 130A: 307–310. 10.1002/ajmg.a.30251 PMC4349519 15378550

[17] Wirth B, Brichta L, Schrank B, Lochmüller H, Blick S, et al (2006) Mildly affected patients with spinal muscular atrophy are partially protected by an increased *SMN2* copy number. Hum Genet 119: 422–428. 16508748 10.1007/s00439-006-0156-7

[18] Swoboda KJ, Prior TW, Scott CB, McNaught TP, Wride MC, et al (2005) Natural history of denervation in SMA: relation to age, *SMN2* copy number and function. Ann Neurol 57: 704–712. 15852397 10.1002/ana.20473 PMC4334582

[19] Elsheikh B, Prior T, Zhang X, Miller R, Kolb SJ, et al (An analysis of disease severity based on *SMN2* copy number in adults with spinal muscular atrophy. Muscle Nerve 40: 652–656. 19760790 10.1002/mus.21350

[20] Tiziano FD, Bertini E, Messina S, Angelozzi C, Pane M, et al (2007) The Hammersmith functional score correlates with the SMN2 copy number: a multicentric study. Neuromuscul Disord 17: 400–403. 17433677 10.1016/j.nmd.2007.02.006

[21] Burghes AHM, Beattie CE (2009) Spinal muscular atrophy: why do low levels of survival motor neuron protein make motor neurons sick? Nat Rev Neurosci 10: 597–609. 19584893 10.1038/nrn2670 PMC2853768

[22] Gabanella F, Carissimi C, Usiello A, Pellizzoni L (2005) The activity of the spinal muscular atrophy protein is regulated during development and cellular differentiation. Hum Mol Genet 14: 3629–3642. 16236758 10.1093/hmg/ddi390

[23] Winkler C, Eggert C, Gradl D, Meister G, Giegerich M, et al (2005) Reduced U snRNP assembly causes motor axon degeneration in an animal model for spinal muscular atrophy. Genes Dev 19: 2320–2330. 16204184 10.1101/gad.342005 PMC1240041

[24] Wan L, Battle DJ, Yong J, Gubitz AK, Kolb SJ, et al (2005) The survival of motor neurons protein determines the capacity for snRNP assembly: biochemical deficiency in spinal muscular atrophy. Mol Cell Biol 25: 5543–5551. 15964810 10.1128/MCB.25.13.5543-5551.2005 PMC1156985

[25] Pagliardini S, Giavizzi A, Setola V, Lizier C, Di Luca M, et al (2000) Subcellular localization and axonal transport of the survival motor neuron (SMN) protein in developing rat spinal cord. Hum Mol Genet 9: 47–56. 10587577 10.1093/hmg/9.1.47

[26] McWhorter ML, Monani UR, Burghes AHM, Beattie CE (2003) Knockdown of the survival motor neuron (Smn) protein in zebrafish causes defects in motor axon outgrowth and pathfinding. J Cell Biol 162: 919–931. 12952942 10.1083/jcb.200303168 PMC1761110

[27] Carrel TL, McWhorter ML, Workman E, Zhang H, Wolstencroft EC, et al (2006) Survival motor neuron function in motor axons is independent of functions required for small nuclear ribonucleoprotein biogenesis. J Neurosci 26: 11014–11022. 17065443 10.1523/JNEUROSCI.1637-06.2006 PMC6674655

[28] Zhang H, Xing L, Rossoll W, Wichterle H, Singer RH, et al (2006) Multiprotein complexes of the survival of motor neuron protein SMN with gemins traffic to neuronal processes and growth cones of motor neurons. J Neurosci 26: 8622–8632. 16914688 10.1523/JNEUROSCI.3967-05.2006 PMC4956918

[29] Mourelatos Z, Abel L, Yong J, Kataoka N, Dreyfuss G (2001) SMN interacts with a novel family of hnRNP and spliceosomal proteins. EMBO J 20: 5443–5452. 11574476 10.1093/emboj/20.19.5443 PMC125643

[30] Rossoll W, Jablonka S, Andreassi C, Kröning AK, Karle K, et al (2003) Smn, the spinal muscular atrophy-determining gene product, modulates axon growth and localization of β-actin mRNA in growth cones of motoneurons. J Cell Biol 163: 801–812. 14623865 10.1083/jcb.200304128 PMC2173668

[31] Bassell GJ, Zhang H, Byrd AL, Femino AM, Singer RH, et al (1998) Sorting of β-actin mRNA and protein to neurites and growth cones in culture. J Neurosci 18: 251–265. 9412505 10.1523/JNEUROSCI.18-01-00251.1998 PMC6793411

[32] Cheever TR, Olson EA, Ervasti JM (2011) Axon regeneration and neuronal function are preserved in motor neurons lacking β-actin *in vivo* . PLoS ONE 6: e17768. 21445349 10.1371/journal.pone.0017768 PMC3062555

[33] Schrank B, Götz R, Gunnersen JM, Ure JM, Toyka KV, et al (1997) Inactivation of the survival motor neuron gene, a candidate gene for human spinal muscular atrophy, leads to massive cell death in early mouse embryos. Proc Natl Acad Sci U S A 94: 9920–9925. 9275227 10.1073/pnas.94.18.9920 PMC23295

[34] Monani UR, Sendtner M, Coovert DD, Parsons DW, Andreassi C, et al (2000) The human centromeric survival motor neuron gene (*SMN2*) rescues embryonic lethality in *Smn^−/−^* mice and results in a mouse with spinal muscular atrophy. Hum Mol Genet 9: 333–339. 10655541 10.1093/hmg/9.3.333

[35] Hsieh-Li HM, Chang JG, Jong YJ, Wu MH, Wang NM, et al (2000) A mouse model for spinal muscular atrophy. Nat Genet 24: 66–70. 10615130 10.1038/71709

[36] Michaud M, Arnoux T, Bielli S, Durand E, Rotrou Y, et al (2010) Neuromuscular defects and breathing disorders in a new mouse model of spinal muscular atrophy. Neurobiol Dis 38: 125–135. 20085811 10.1016/j.nbd.2010.01.006

[37] Wichterle H, Lieberam I, Porter JA, Jessell TM (2002) Directed differentiation of embryonic stem cells into motor neurons. Cell 110: 385–397. 12176325 10.1016/s0092-8674(02)00835-8

[38] Miles GB, Yohn DC, Wichterle H, Jessell TM, Rafuse VF, et al (2004) Functional properties of motoneurons derived from mouse embryonic stem cells. J Neurosci 24: 7848–7858. 15356197 10.1523/JNEUROSCI.1972-04.2004 PMC6729934

[39] Makhortova NR, Hayhurst M, Cerqueira A, Sinor-Anderson AD, Zhao WN, et al (2011) A screen for regulators of survival of motor neuron protein levels. Nat Chem Biol 7: 544–552. 21685895 10.1038/nchembio.595 PMC3236614

[40] Anderson KN, Baban D, Oliver PL, Potter A, Davies KE (2004) Expression profiling in spinal muscular atrophy reveals an RNA binding protein deficit. Neuromuscul Disord 14: 711–722. 15482955 10.1016/j.nmd.2004.08.009

[41] Zhang Z, Lotti F, Dittmar K, Younis I, Wan L, et al (2008) SMN deficiency causes tissue-specific perturbations in the repertoire of snRNAs and widespread defects in splicing. Cell 133: 585–600. 18485868 10.1016/j.cell.2008.03.031 PMC2446403

[42] Murray LM, Lee S, Bäumer D, Parson SH, Talbot K, et al (2010) Pre-symptomatic development of lower motor neuron connectivity in a mouse model of severe spinal muscular atrophy. Hum Mol Genet 19: 420–433. 19884170 10.1093/hmg/ddp506

[43] Sultan M, Schulz MH, Richard H, Magen A, Klingenhoff A, et al (2008) A global view of gene activity and alternative splicing by deep sequencing of the human transcriptome. Science 321: 956–960. 18599741 10.1126/science.1160342

[44] Wang Z, Gerstein M, Snyder M (2009) RNA-Seq: a revolutionary tool for transcriptosomics. Nat Rev Genet 10: 57–63. 19015660 10.1038/nrg2484 PMC2949280

[45] McGovern VL, Gavrilina TO, Beattie CE, Burghes AHM (2008) Embryonic motor axon development in the severe SMA mouse. Hum Mol Genet 17: 2900–2909. 18603534 10.1093/hmg/ddn189 PMC2722893

[46] Wu CY, Whye D, Glazewski L, Choe L, Kerr D, et al (2011) Proteomic assessment of a cell model of spinal muscular atrophy. BMC Neurosci 12: 25. 21385431 10.1186/1471-2202-12-25 PMC3063191

[47] Wu CY, Whye D, Mason RW, Wang W (2012) Efficient differentation of mouse embryonic stem cells into motor neurons. J Vis Exp 3813. 10.3791/3813 PMC3471293 22711008

[48] Butchbach MER, Edwards JD, Schussler KR, Burghes AHM (2007) A novel method for oral delivery of compounds to the neonatal SMNΔ7 model of spinal muscular atrophy. J Neurosci Methods 161: 285–290. 17161463 10.1016/j.jneumeth.2006.11.002 PMC2699996

[49] Barrett T, Wilhite SE, Ledoux P, Evangelista C, Kim IF, et al (2013) NCBI GEO: archive for functional genomics data sets–update. Nucleic Acids Res 41: D991–D995. 23193258 10.1093/nar/gks1193 PMC3531084

[50] Martin M (2011) Cutadapt removes adapter sequences from high-throughput sequencing reads. EMBNet Journal 17: 10–12.

[51] Trapnell C, Pachter L, Salzberg SL (2009) TopHat: discovering splice junctions with RNA-Seq. Bioinformatics 25: 1105–1111. 19289445 10.1093/bioinformatics/btp120 PMC2672628

[52] Trapnell C, Williams BA, Pertea G, Mortazavi A, Kwan G, et al (2010) Transcript assembly and quantification by RNA-Seq reveals unannotated transcripts and isoform switching during cell differentiation. Nat Biotechnol 28: 511–515. 20436464 10.1038/nbt.1621 PMC3146043

[53] Trapnell C, Roberts A, Goff L, Pertea G, Kim D, et al (2012) Differential gene and transcript expression analysis of RNA-seq experiments with TopHat and Cufflinks. Nat Protoc 7: 562–578. 22383036 10.1038/nprot.2012.016 PMC3334321

[54] Juknat A, Pietr M, Kozela E, Rimmerman N, Levy R, et al (2013) Microarray and pathway analysis reveal distinct mechanisms underlying cannabinoid-mediated modulation of LPS-induced activation of BV-2 microglial cells. PLoS ONE 8: e61462. 23637839 10.1371/journal.pone.0061462 PMC3634783

[55] Krämer A, Green J, Pollard Jr J, Tugendreich S (2014) Causal analysis approaches in Ingenuity Pathway Analysis. Bioinformatics 30: 523–530. 24336805 10.1093/bioinformatics/btt703 PMC3928520

[56] Schmittgen TD, Livak KJ (2008) Analyzing real-time PCR data by the comparative C_T_ method. Nat Protoc 3: 1101–1108. 18546601 10.1038/nprot.2008.73

[57] Vandesompele J, De Preter K, Pattyn F, Poppe B, Van Roy N, et al (2002) Accurate normalization of real-time quantitative RT-PCR data by geometric averaging of multiple internal control genes. Genome Biol 3: research0034.1–research0034.11. 12184808 10.1186/gb-2002-3-7-research0034 PMC126239

[58] Arber S, Han B, Mendelsohn M, Smith M, Jessell TM, et al (1999) Requirement for the homeobox gene Hb9 in the consolidation of motor neuron identity. Neuron 23: 659–674. 10482234 10.1016/s0896-6273(01)80026-x

[59] Pfaff SL, Mendelsohn M, Stewart CL, Edlund T, Jessell TM (1996) Requirement for LIM homeobox gene Isl1 in motor neuron generation reveals a motor neuron-dependent step in interneuron differentiation. Cell 84: 309–320. 8565076 10.1016/s0092-8674(00)80985-x

[60] Fang Z, Cui X (2011) Design and validation issues in RNA-seq experiments. Brief Bioinform 12: 280–287. 21498551 10.1093/bib/bbr004

[61] Jablonka S, Schrank B, Kralewski M, Rossoll W, Sendtner M (2000) Reduced survival motor neuron (*Smn*) gene dose in mice leads to motor neuron degeneration: an animal model for spinal muscular atrophy type III. Hum Mol Genet 9: 341–346. 10655542 10.1093/hmg/9.3.341

[62] Garcia EL, Lu Z, Meers MP, Praveen K, Matera AG (2013) Developmental arrest of Drosophila survival motor neuron (Smn) mutants accounts for differences in expression of minor intron-containing genes. RNA 19: 1510–1516. 24006466 10.1261/rna.038919.113 PMC3851718

[63] See K, Yadav P, Giegerich M, Cheong PS, Graf M, et al (2014) SMN deficiency alters Nrxn2 expression and splicing in zebrafish and mouse models of spinal muscular atrophy. Hum Mol Genet 23: 1754–1770. 24218366 10.1093/hmg/ddt567

[64] Zhang Z, Pinto AM, Wan L, Wang W, Berg MG, et al (2013) Dysregulation of synaptogenesis genes antecedes motor neuron pathology in spinal muscular atrophy. Proc Natl Acad Sci U S A 110: 19348–19353. 24191055 10.1073/pnas.1319280110 PMC3845193

[65] Park GH, Maneo-Hikichi Y, Awano T, Landmesser LT, Monani UR (2010) Reduced survival of motor neuron (SMN) protein in motor neuronal progenitors functions cell autonomously to cause spinal muscular atrophy in model mice expressing the human centromeric (*SMN2*) gene. J Neurosci 30: 12005–12019. 20826664 10.1523/JNEUROSCI.2208-10.2010 PMC2944776

[66] Martinez TL, Kong L, Wang X, Osborne MA, Crowder ME, et al (2012) Survival motor neuron protein in motor neurons determines synaptic integrity in spinal muscular atrophy. J Neurosci 32: 8703–8715. 22723710 10.1523/JNEUROSCI.0204-12.2012 PMC3462658

[67] Gogliotti RG, Quinlan KA, Barlow CB, Heier CR, Heckman CJ, et al (2012) Motor neuron rescue in spinal muscular atrophy mice demonstrates that sensory-motor defects are a consequence, not a cause, of motor neuron dysfunction. J Neurosci 32: 3818–3829. 22423102 10.1523/JNEUROSCI.5775-11.2012 PMC3679185

[68] Gavrilina TO, McGovern VL, Workman E, Crawford TO, Gogliotti RG, et al (2008) Neuronal SMN expression corrects spinal muscular atrophy in severe SMA mice while muscle specific SMN expression has no phenotypic effect. Hum Mol Genet 17: 1063–1075. 18178576 10.1093/hmg/ddm379 PMC2835541

[69] Hua Y, Sahashi K, Rigo F, Hung G, Horev G, et al (2011) Peripheral SMN restoration is essential for long-term rescue of a severe spinal muscular atrophy mouse model. Nature 478: 123–126. 21979052 10.1038/nature10485 PMC3191865

[70] Passini MA, Bu J, Roskelley EM, Richards AM, Sardi SP, et al (2010) CNS-targeted gene therapy improves survival and motor function in a mouse model of spinal muscular atrophy. J Clin Invest 120: 1253–1264. 20234094 10.1172/JCI41615 PMC2846065

[71] Foust KD, Wang X, McGovern VL, Braun L, Bevan AK, et al (2010) Rescue of the spinal muscular atrophy phenotype in a mouse model by early postnatal delivery of *SMN* . Nat Biotechnol 28: 271–274. 20190738 10.1038/nbt.1610 PMC2889698

[72] Butchbach MER, Singh J, Þorsteindóttir M, Saieva L, Slominski E, et al (2010) Effects of 2,4-diaminoquinazoline derivatives on SMN expression and phenotype in a mouse model for spinal muscular atrophy. Hum Mol Genet 19: 454–467. 19897588 10.1093/hmg/ddp510 PMC2798721

[73] Van Meerbeke JP, Gibbs RM, Plasterer HL, Miao W, Feng Z, et al (2013) The DcpS inhibitor RG3039 improves motor function in SMA mice. Hum Mol Genet 22: 4074–4083. 23727836 10.1093/hmg/ddt257 PMC3781637

[74] Gogliotti RG, Cardona H, Singh J, Bail S, Emery C, et al (2013) The DcpS inhibitor RG3039 improves survival, function and motor unit pathologies in two SMA mouse models. Hum Mol Genet 22: 4084–4101. 23736298 10.1093/hmg/ddt258 PMC3781638

[75] Azzouz M, Le T, Ralph GS, Walmsley L, Monani UR, et al (2004) Lentivector-mediated SMN replacement in a mouse model of spinal muscular atrophy. J Clin Invest 114: 1726–1731. 15599397 10.1172/JCI22922 PMC535071

[76] Dominguez E, Marais T, Chatauret N, Benkhelifa-Ziyyat S, Duque S, et al (2011) Intravenous scAAV9 delivery of a codon-optimized SMN1 sequence rescues SMA mice. Hum Mol Genet 20: 681–693. 21118896 10.1093/hmg/ddq514

[77] Valori CF, Ning K, Wyles M, Mead RJ, Grierson AJ, et al (2010) Systemic delivery of scAAV9 expressin SMN prolongs survival in a model of spinal muscular atrophy. Sci Transl Med 2: 35ra42. 10.1126/scitranslmed.3000830 20538619

[78] Boyer LA, Lee TI, Cole MF, Johnstone SE, Levine SS, et al (2005) Core transcriptional regulatory circuitry in human embryonic stem cells. Cell 122: 947–956. 16153702 10.1016/j.cell.2005.08.020 PMC3006442

[79] Redmond LC, Dumur CI, Archer KJ, Grayson DR, Haar JL, et al (2011) Krüppel-like factor 2 regulated gene expression in mouse embryonic yolk sac erythroid cells. Blood Cells Mol Dis 47: 1–11. 21530336 10.1016/j.bcmd.2011.03.002 PMC3150518

[80] Lim LS, Loh YH, Zhang W, Li Y, Chen X, et al (2007) Zic3 is required for maintenance of pluripotency in embryonic stem cells. Mol Biol Cell 18: 1348–1358. 17267691 10.1091/mbc.E06-07-0624 PMC1838990

[81] Zhang W, Walker E, Tamplin OJ, Rossant J, Stanford WL, et al (2006) Zfp206 regulates ES cell gene expression and differentiation. Nucleic Acids Res 34: 4780–4790. 16971461 10.1093/nar/gkl631 PMC1635278

[82] Kim SK, Suh MR, Yoon HS, Lee JB, Oh SK, et al (2005) Identification of developmental pluripotency associated 5 expression in human pluripotent stem cells. Stem Cells (Dayt) 23: 458–462. 15790765 10.1634/stemcells.2004-0245

[83] Masui S, Ohtsuka S, Yagi R, Takahashi K, Ko MSH, et al (2008) Rex1/Zfp42 is dispensable for pluripotency in mouse ES cells. BMC Dev Biol 8: 45. 18433507 10.1186/1471-213X-8-45 PMC2386458

[84] Papetti M, Aughenlicht LH (2010) MYBL2, a link between proliferation and differentiation in maturing colon epithelial cells. J Cell Physiol 226: 785–791. 10.1002/jcp.22399 PMC3012743 20857481

[85] Boyne LJ, Fischer I, Shea TB (1996) Role of vimentin in early stages of neuritogenesis in cultured hippocampal neurons. Int J Dev Neurosci 14: 739–748. 8960981 10.1016/s0736-5748(96)00053-6

[86] Briscoe J, Sussel L, Serup P, Hartigan-O'Connor D, Jessell TM, et al (1999) Homeobox gene *Nkx2.2* and specification of neuronal identity by graded Sonic hedgehog signaling. Nature 398: 622–627. 10217145 10.1038/19315

[87] Pierfelice T, Alberi L, Gaiano N (2011) Notch in the vertebrate nervous system: an old dog with new tricks. Neuron 69: 855. 10.1016/j.neuron.2011.02.031 21382546

[88] Caraballo-Miralles V, Cardona-Rossinyol A, Garcera A, Torres-Benito L, Soler RM, et al (2013) Notch signaling pathway is activated in motoneurons of spinal muscular atrophy. Int J Mol Sci 14: 11424–11437. 23759991 10.3390/ijms140611424 PMC3709740

[89] Dencker L, Annerwall E, Busch C, Erikkson U (1990) Localization of specific retinoid-binding sites and expression of cellular retinoic-acid-binding protein (CRABP) in the early mouse embryo. Development 110: 343–352. 1966832 10.1242/dev.110.2.343

[90] Perez-Castro AV, Toth-Rogler LE, Wei L, Nguyen-Huu MC (1989) Spatial and temporal pattern of expression of the cellular retinoic acid-binding protein and the cellular retinol-binding protein during mouse embryogenesis. Proc Natl Acad Sci U S A 86: 8813–8817. 2554331 10.1073/pnas.86.22.8813 PMC298380

[91] Hosler BA, LaRosa GJ, Grippo JF, Gudas LJ (1989) Expression of REX-1, a gene containing zinc finger motifs, is rapidly reduced by retinoic acid in F9 teratocarcinoma cells. Mol Cell Biol 9: 5623–5629. 2511439 10.1128/mcb.9.12.5623-5629.1989 PMC363733

[92] Appel B, Eisen JS (2003) Retinoids run rampant: multiple roles during spinal cord and motor neuron development. Neuron 40: 461–464. 14642271 10.1016/s0896-6273(03)00688-3

[93] Diez del Corral R, Olivera-Martinez I, Goriely A, Gale E, Maden M, et al (2003) Opposing FGF and retinoid pathways control ventral nerve pattern, neuronal differentiation and segmentation during body axis formation. Neuron 40: 65–69. 14527434 10.1016/s0896-6273(03)00565-8

[94] Novitch BG, Wichterle H, Jessell TM, Sockanathan S (2003) A requirement for retinoic acid-mediated transcriptional activation in ventral neural patterning and motor neuron specification. Neuron 40: 81–95. 14527435 10.1016/j.neuron.2003.08.006

[95] Liu H, Shafey D, Moores JN, Kothary R (2010) Neurodevelopmental consequences of Smn depletion in a mouse model of spinal muscular atrophy. J Neurosci Res 88: 111–122. 19642194 10.1002/jnr.22189

[96] Ebert AD, Yu J, Rose Jr FF, Mattis VB, Lorson CL, et al (2009) Induced pluripotent stem cells from a spinal muscular atrophy patient. Nature 457: 277–280. 19098894 10.1038/nature07677 PMC2659408

[97] Chang T, Zheng W, Tsark W, Bates S, Huang H, et al (2011) Phenotypic rescue of induced pluripotent stem cell-derived motoneurons of a spinal muscular atrophy patient. Stem Cells (Dayt) 29: 2090–2093. 21956898 10.1002/stem.749

[98] Corti S, Nizzardo M, Simone C, Falcone M, Nardini M, et al (2012) Genetic correction of human induced pluripotent stem cells from patients with spinal muscular atrophy. Sci Transl Med 4: 165ra162. 10.1126/scitranslmed.3004108 PMC4722730 23253609

[99] Sareen D, Ebert AD, Heins BM, McGivern JV, Ornelas L, et al (2012) Inhibition of apoptosis blocks human motor neuron cell death in a stem cell model of spinal muscular atrophy. PLoS ONE 7: e39113. 22723941 10.1371/journal.pone.0039113 PMC3378532

[100] Le TT, Pham LT, Butchbach MER, Zhang HL, Monani UR, et al (2005) SMNΔ7, the major product of the centromeric survival motor neuron gene (*SMN2*), extends survival in mice with spinal muscular atrophy and associates with full-length SMN. Hum Mol Genet 14: 845–857. 15703193 10.1093/hmg/ddi078

[101] Kariya S, Park GH, Maeno-Hikichi Y, Leykekhman O, Lutz C, et al (2008) Reduced SMN protein impairs maturation of the neuromuscular junctions in mouse models of spinal muscular atrophy. Hum Mol Genet 17: 2552–2569. 18492800 10.1093/hmg/ddn156 PMC2722888

[102] Chang HCH, Dimlich DN, Yokokura T, Mukherjee A, Kankel MW, et al (2008) Modeling spinal muscular atrophy in Drosophila. PLoS ONE 3: e3209. 18791638 10.1371/journal.pone.0003209 PMC2527655

[103] Kong L, Wang X, Choe DW, Polley M, Burnett BG, et al (2009) Impaired synaptic vesicle release and immaturity of neuromuscular junctions in spinal muscular atrophy mice. J Neurosci 29: 842–851. 19158308 10.1523/JNEUROSCI.4434-08.2009 PMC2746673

